# Effects of repeat prenatal corticosteroids given to women at risk of preterm birth: An individual participant data meta-analysis

**DOI:** 10.1371/journal.pmed.1002771

**Published:** 2019-04-12

**Authors:** Caroline A. Crowther, Philippa F. Middleton, Merryn Voysey, Lisa Askie, Sasha Zhang, Tanya K. Martlow, Fariba Aghajafari, Elizabeth V. Asztalos, Peter Brocklehurst, Sourabh Dutta, Thomas J. Garite, Debra A. Guinn, Mikko Hallman, Pollyanna Hardy, Men-Jean Lee, Kimberley Maurel, Premasish Mazumder, Cindy McEvoy, Kellie E. Murphy, Outi M. Peltoniemi, Elizabeth A. Thom, Ronald J. Wapner, Lex W. Doyle, the PRECISE Group

**Affiliations:** 1 Liggins Institute, University of Auckland, Auckland, New Zealand; 2 Robinson Research Institute, Discipline of Obstetrics and Gynaecology, School of Medicine, The University of Adelaide, Adelaide, Australia; 3 Healthy Mothers Babies and Children, South Australian Health and Medical Research Institute, Adelaide, Australia; 4 Nuffield Department of Primary Care Health Sciences and Department of Paediatrics, University of Oxford, Oxford, United Kingdom; 5 NHMRC Clinical Trials Centre, University of Sydney, Sydney, Australia; 6 Department of Family Medicine, Cumming School of Medicine, University of Calgary, Calgary, Alberta, Canada; 7 Department of Paediatrics and Obstetrics/Gynecology, University of Toronto, Toronto, Ontario, Canada; 8 Birmingham Clinical Trials Unit, Institute of Applied Health Research, University of Birmingham, Birmingham, United Kingdom; 9 Division of Neonatology, Department of Pediatrics, Postgraduate Institute of Medical Education and Research, Chandigarh, India; 10 Mednax Inc., Sunrise, Florida, United States of America; 11 Kalispell Regional Health Care, Kalispell, Montana, United States of America; 12 Department of Paediatrics, University of Oulu, Oulu, Finland; 13 John A Burns School of Medicine, University of Hawaii, Honolulu, Hawaii, United States of America; 14 Department of Pediatrics, Oregon Health and Science University, Portland, Oregon, United States of America; 15 The Biostatistics Center, George Washington University, Washington, DC, United States of America; 16 Department of Obstetrics and Gynecology, Division of Maternal Fetal Medicine, Columbia University Medical Center, New York, New York, United States of America; 17 Department of Obstetrics and Gynaecology, The Royal Women’s Hospital, University of Melbourne, Melbourne, Australia; 18 Clinical Sciences, Murdoch Children’s Research Institute, Parkville, Victoria, Australia; 19 Department of Paediatrics, University of Melbourne, Parkville, Victoria, Australia; University of Manchester, UNITED KINGDOM

## Abstract

**Background:**

Infants born preterm compared with infants born at term are at an increased risk of dying and of serious morbidities in early life, and those who survive have higher rates of neurological impairments. It remains unclear whether exposure to repeat courses of prenatal corticosteroids can reduce these risks. This individual participant data (IPD) meta-analysis (MA) assessed whether repeat prenatal corticosteroid treatment given to women at ongoing risk of preterm birth in order to benefit their infants is modified by participant or treatment factors.

**Methods and findings:**

Trials were eligible for inclusion if they randomised women considered at risk of preterm birth who had already received an initial, single course of prenatal corticosteroid seven or more days previously and in which corticosteroids were compared with either placebo or no placebo. The primary outcomes for the infants were serious outcome, use of respiratory support, and birth weight z-scores; for the children, they were death or any neurosensory disability; and for the women, maternal sepsis. Studies were identified using the Cochrane Pregnancy and Childbirth search strategy. Date of last search was 20 January 2015. IPD were sought from investigators with eligible trials. Risk of bias was assessed using criteria from the Cochrane Collaboration. IPD were analysed using a one-stage approach.

Eleven trials, conducted between 2002 and 2010, were identified as eligible, with five trials being from the United States, two from Canada, and one each from Australia and New Zealand, Finland, India, and the United Kingdom. All 11 trials were included, with 4,857 women and 5,915 infants contributing data. The mean gestational age at trial entry for the trials was between 27.4 weeks and 30.2 weeks. There was no significant difference in the proportion of infants with a serious outcome (relative risk [RR] 0.92, 95% confidence interval [CI] 0.82 to 1.04, 5,893 infants, 11 trials, *p* = 0.33 for heterogeneity). There was a reduction in the use of respiratory support in infants exposed to repeat prenatal corticosteroids compared with infants not exposed (RR 0.91, 95% CI 0.85 to 0.97, 5,791 infants, 10 trials, *p* = 0.64 for heterogeneity). The number needed to treat (NNT) to benefit was 21 (95% CI 14 to 41) women/fetus to prevent one infant from needing respiratory support. Birth weight z-scores were lower in the repeat corticosteroid group (mean difference −0.12, 95%CI −0.18 to −0.06, 5,902 infants, 11 trials, *p* = 0.80 for heterogeneity). No statistically significant differences were seen for any of the primary outcomes for the child (death or any neurosensory disability) or for the woman (maternal sepsis). The treatment effect varied little by reason the woman was considered to be at risk of preterm birth, the number of fetuses in utero, the gestational age when first trial treatment course was given, or the time prior to birth that the last dose was given. Infants exposed to between 2–5 courses of repeat corticosteroids showed a reduction in both serious outcome and the use of respiratory support compared with infants exposed to only a single repeat course. However, increasing numbers of repeat courses of corticosteroids were associated with larger reductions in birth z-scores for weight, length, and head circumference. Not all trials could provide data for all of the prespecified subgroups, so this limited the power to detect differences because event rates are low for some important maternal, infant, and childhood outcomes.

**Conclusions:**

In this study, we found that repeat prenatal corticosteroids given to women at ongoing risk of preterm birth after an initial course reduced the likelihood of their infant needing respiratory support after birth and led to neonatal benefits. Body size measures at birth were lower in infants exposed to repeat prenatal corticosteroids. Our findings suggest that to provide clinical benefit with the least effect on growth, the number of repeat treatment courses should be limited to a maximum of three and the total dose to between 24 mg and 48 mg.

## Abstract 2

In an individual participant data meta-analysis, Caroline Anne Crowther and colleagues assess the effects of repeat prenatal corticosteroid treatment given to women at on-going risk of preterm birth.

## Author summary

**Why was this study done?:**

- Effective therapies that can reduce the risk of infants born preterm having respiratory and other health problems early in life and prevent later neurological impairments are needed. A single course of prenatal corticosteroids prior to expected or planned preterm birth remains the gold standard treatment to reduce these risks.
- If birth does not occur, it has been unclear whether giving women who remain at high risk of preterm birth a repeat course or courses of prenatal corticosteroids can similarly reduce the risks for the infant.
- The aims of the Prenatal Repeat Corticosteroid International IPD-MA Study: assessing the effects using the best level of Evidence (PRECISE) individual participant data meta-analysis (IPD-MA) were to assess the effect of repeat prenatal corticosteroids when given to women at high, ongoing risk of preterm birth on important clinical outcomes and whether treatment effects varied depending on participant and treatment factors.

**What did the researchers do and find?:**

- Eleven randomised trials with 4,857 women and 5,915 infants were identified as including women at ongoing risk of preterm birth after an initial course of prenatal corticosteroids who were allocated repeat prenatal corticosteroids or placebo or no placebo treatment and in which outcomes for the infant were reported and included.
- Overall, repeat prenatal corticosteroids given to women at ongoing risk of preterm birth after an initial course did not reduce the risk of serious health outcomes for the infant but did reduce the need for respiratory support, although birth weight z-scores were reduced. Exposure to more repeat courses and greater total dose of corticosteroids was associated with a reduction in serious outcome and less need for respiratory support for the infant but also greater reduction in body size at birth. There were no differences in rates of maternal sepsis. At childhood follow-up, no differences in neurodevelopment were seen between those exposed to repeat prenatal corticosteroids and those not, although weight z-scores and systolic blood pressure measurements were lower in the exposed group.
- Treatment effects varied little by reason for preterm birth, the number of fetuses in utero, the gestational age when repeat treatment was given, or the time prior to birth that the last dose was given.

**What do these findings mean?:**

- Repeat prenatal corticosteroids given to women at ongoing risk of preterm birth after an initial course reduce the likelihood of their infant needing respiratory support after birth and lead to other respiratory benefits.
- To provide clinical benefit with the least effect on growth, the number of repeat treatment courses should be limited if possible. We suggest a maximum of three repeat treatments and a total dose of between 24 mg and 48 mg.
- Reductions in weight z-scores and systolic blood pressure seen at childhood follow-up are of uncertain clinical importance and warrant further study.
- Adoption of repeat prenatal corticosteroid treatment used in this way could lead to both significant health benefits for infants at ongoing risk of preterm birth after exposure to an initial course of corticosteroids while minimising any reduction in body size.

## Introduction

Respiratory distress syndrome, caused by surfactant deficiency, is a common complication of preterm birth and a major cause of early neonatal mortality and morbidity [1]. Infants born preterm often require respiratory support, with significant numbers requiring assisted ventilation and treatment with exogenous surfactant [1]. Children born preterm who survive have a higher risk of subsequent long-term neurodevelopmental impairments such as cerebral palsy, cognitive dysfunction, blindness, and deafness than do children born at term [2,3]. The global burden is substantial, with estimates suggesting up to 8% of preterm infants have neurological impairments [4]. The personal and emotional costs for affected individuals and their families are high, as are the immediate and long-term societal costs [2,3,5,6]. Effective antenatal treatments that can reduce the morbidities of preterm birth are clearly needed. A single course of antenatal corticosteroids remains the ‘gold standard’ antenatal intervention to reduce the risk of fetal and neonatal mortality related to preterm birth, but residual morbidity remains a problem [7].

Use of repeat prenatal corticosteroids for women who remain at risk of preterm birth to prevent neonatal morbidity was first suggested in the early 1970s [8]. The Cochrane systematic review assessing the use of repeat prenatal corticosteroids for women at risk of preterm birth to prevent neonatal disease shows their use results in a clinically important reduction in both the risk of respiratory distress syndrome (risk ratio 0.83, 95% confidence intervals [CIs] 0.75 to 0.91; eight trials; 3,206 infants; number needed to treat [NNT] 17, 95% CI 11 to 32) and of serious infant outcome (risk ratio 0.84, 95% CI 0.75 to 0.94; seven trials; 5,094 infants; NNT 30, 95% CI 19 to 79) [9]. These neonatal benefits support the use of repeat dose(s) of prenatal corticosteroids for women who have received an initial course of prenatal corticosteroids 7 or more days previously and who remain at risk of preterm birth. However, the aggregate review is unable to provide important details needed by guideline developers [10], clinicians, and pregnant women and their families, notably, whether repeat prenatal corticosteroids are more effective in some women by reason of their risk of preterm birth; what is the best gestational age for administration for maximal benefit; and what dose, number of repeat doses, and timing prior to birth are optimal.

To address these questions, the Prenatal Repeat Corticosteroid International IPD-MA Study: assessing the effects using the best level of Evidence (PRECISE) Group was formed to undertake an individual participant data (IPD) meta-analysis (MA) of the data from eligible trials. This approach has the advantages of allowing for exploration of interactions between treatment and characteristics at a participant level and enables examination of differential treatment effects between subgroups [11,12,13].

## Materials and methods

Results are reported using the Preferred Items for Reporting for Systematic Review and Meta-analysis of Individual Participant Data (PRISMA-IPD Statement) checklist [14] (S1 Text). The IPD-MA followed the published protocol [15] (S2 Text). The Child Youth and Women’s Health Service Research Ethics Committee, South Australia, Australia, approved the study (REC2334/12/13). The included trials had received country- and site-specific ethical review, with individual participants providing written, informed consent.

### Specific objectives

The objectives of the PRECISE IPD-MA were to assess, using IPD methods and MAs, the effects on important clinical outcomes of repeat prenatal corticosteroid treatment given to women at risk of preterm birth to benefit their infants, both short-term and long-term, and whether the treatment effects differed by the following prespecified participant and treatment characteristics [15]: the reason the women were at risk of preterm birth (e.g., antepartum haemorrhage, preterm prelabour rupture of the membranes, preterm labour, hypertensive disease of pregnancy); the gestational age when the repeat prenatal corticosteroids were given; and the dose, number of repeat doses, and timing prior to birth that treatment was given.

### Eligibility criteria

Trials were eligible if they randomised women considered at risk of preterm birth (<37 weeks’ gestation), who had already received a single course of prenatal corticosteroids seven or more days previously, to either corticosteroids—administered to the women intravenously, intramuscularly, or orally—or placebo/no placebo, and if they reported data on one or more of the prespecified outcomes. Quasirandom study designs were not eligible.

### Identifying studies: Information sources and search strategy

The search strategy developed by Cochrane Pregnancy and Childbirth was used [16]. This identified trials from monthly searching of the Cochrane Central Register of Controlled Trials (CENTRAL), weekly searches of MEDLINE, monthly searches of CINAHL (EBSCO), hand searches of 30 journals and the proceedings of major conferences, and weekly current awareness alerts for a further 44 journals plus monthly BioMed Central email alerts.

Search terms used were [repeat or multiple] and [antenatal or prenatal] and [corticosteroid* or steroid* or glucocorticoid* or betamethason* or dexamethason* or hydrocortison*]. Date of last search was 20 January 2015. The WHO/CTRP portal was accessed to identify any recently completed or ongoing trials. There was no language restriction on searches. Trialists within the PRECISE Group were asked if they knew of any unpublished or other trials.

### Study selection processes

Eligibility of identified trials was assessed independently and unblinded by two members of the PRECISE Project Team (PFM and TKM). Any differences regarding eligibility were resolved by discussion.

### Data collection processes and data items

The Chairperson of the PRECISE Project Team (CAC) contacted the investigators of all eligible studies to invite them to join the PRECISE Trialist Group and to include IPD data from their trial in the IPD-MA. Prespecified variables were developed and defined by the PRECISE Trialist Group. Investigators were asked if these variables were available or could be derived for their study, and a coding system was developed.

Deidentified data were collected on all randomised women. These included baseline data for descriptive purposes and analyses (reason at risk of preterm birth, gestational age at trial entry, plurality of the pregnancy, expected date of birth) and details of the intervention planned and given (date of randomisation, allocated intervention, type and dose of antenatal corticosteroids given, mode of administration, number and frequency of repeat courses given) together with the infant, childhood, and maternal outcomes to allow the planned analyses.

The data from the individual trials were recoded as needed and stored in a secure database only accessible by the PRECISE Data Management Group. The individual trialists were asked to verify their coded data prior to analyses. Data were checked for internal consistency, extreme or missing values, errors, and consistency with published reports. Randomisation methods and intervention details were cross-checked against trial protocols, clinical record forms, and published reports for all trials. The PRECISE biostatistician (MV), project coordinator (TKM), and data manager (SZ) liaised with the trialists to check any inconsistencies and missing data. The final IPD dataset prepared from each trial was sent to the trialist for verification before being included in the full PRECISE dataset. Data items collected are published elsewhere [15] (S2 Text).

### Risk of bias assessment in individual studies

Risk of bias for each study was assessed using the Cochrane Collaboration risk of bias tool [17] by two members of the PRECISE Project Team (PFM and TKM) independently, with differences resolved by discussion. Each study was judged to have high, low, or unclear risk of bias for random sequence generation (checking for possible selection bias), allocation concealment (checking for possible selection bias), blinding of participants and personnel (checking for possible performance bias), blinding of outcome assessment (checking for possible detection bias), incomplete outcome data (possible attrition bias due to the amount, nature, and handling of incomplete outcome data), selective reporting (checking for possible reporting bias), and other bias (checking for bias due to problems not already covered). The magnitude and direction of the bias and whether it was considered likely to affect the findings were assessed. If any aspect was unclear, additional information was sought from the trialists.

### Specification of outcomes and effect measures

The primary prespecified outcomes for the infant were serious outcome (defined by the PRECISE Group as any of death [fetus, neonatal, infant, or child], severe respiratory disease as defined by the trialists, grade 3 or 4 intraventricular haemorrhage, chronic lung disease [oxygen dependent at 36 weeks postmenstrual age], definite necrotising enterocolitis, stage 3 or worse retinopathy of prematurity in the better eye, or cystic periventricular leucomalacia); use of respiratory support (defined as mechanical ventilation or continuous positive airways pressure, ECMO, oscillatory ventilation, or any other form of assisted ventilation); and birth weight z-scores.

The primary prespecified outcome for the child was death or any neurosensory disability at childhood follow-up (defined as developmental delay or intellectual impairment [developmental quotient or intelligence quotient more than one standard deviation below the mean], cerebral palsy [abnormality of tone with motor dysfunction], blindness ([corrected visual acuity worse than 6/60 in the better eye], or deafness [hearing loss requiring amplification or worse]).

The primary prespecified outcome for the woman was maternal sepsis (defined as chorioamnionitis during labour, pyrexia after trial entry requiring the use of antibiotics, puerperal sepsis, or intrapartum fever requiring the use of antibiotics or postnatal pyrexia).

Secondary outcomes were prespecified in the PRECISE IPD-MA protocol [15] (S2 Text).

### Synthesis methods and additional analyses

The statistical analysis plan was prepared by the PRECISE Data Management Group and agreed by the PRECISE Trialist Group. One-step IPD-MA analyses were conducted on the checked IPD from all the trials. All randomised participants with outcome data available were included in the analyses, performed on an intention-to-treat basis by treatment allocation at randomisation.

Binary outcomes were analysed using log binomial regression models with results presented as RRs with 95% CIs and associated two-sided *p* values. Continuous outcomes were analysed using linear regression models, and results were presented as differences in means with 95% CI and two-sided *p* values. Correlations between outcomes due to multiple births were taken into account using generalised estimating equations (GEEs) for infant and child outcomes. Differences in treatment effect between prespecified subgroups were assessed by testing a treatment-by-subgroup interaction term within the model. A treatment-by-trial interaction term was included in the model to assess heterogeneity of treatment effect across trials. When excessive statistical heterogeneity in treatment effect or inconsistency across trials was detected, then the rationale for combining trials was questioned and the source of heterogeneity explored.

For growth outcomes, z-scores were calculated at birth, hospital discharge, and follow-up based on WHO Child Growth Standards and British 1990 reference data, reanalysed 2009 [18,19]. For composite outcomes, some trials did not collect all variables used to define the composite. Analysis of composite outcomes included all trials that collected at least one variable used to define the composite. Participants were classified as having the outcome if they experienced any of the events contributing to the composite for which data were available and classified as not having the outcome otherwise. Statistical significance was assessed at the *p* < 0.05 level. NNT was calculated as the inverse of the absolute risk reduction (ARR): NNT = 1/ARR, where ARR = Control Event Rate minus Experimental Event Rate.

Analyses presented do not correspond to published estimates from individual trials in every case because trialists may have defined outcomes differently or prespecified different criteria for inclusion or exclusion of data from their analysis. Discrepancies between published estimates and PRECISE estimates do not imply that one or the other is incorrect.

## Results

### Study characteristics

**Fig 1:**
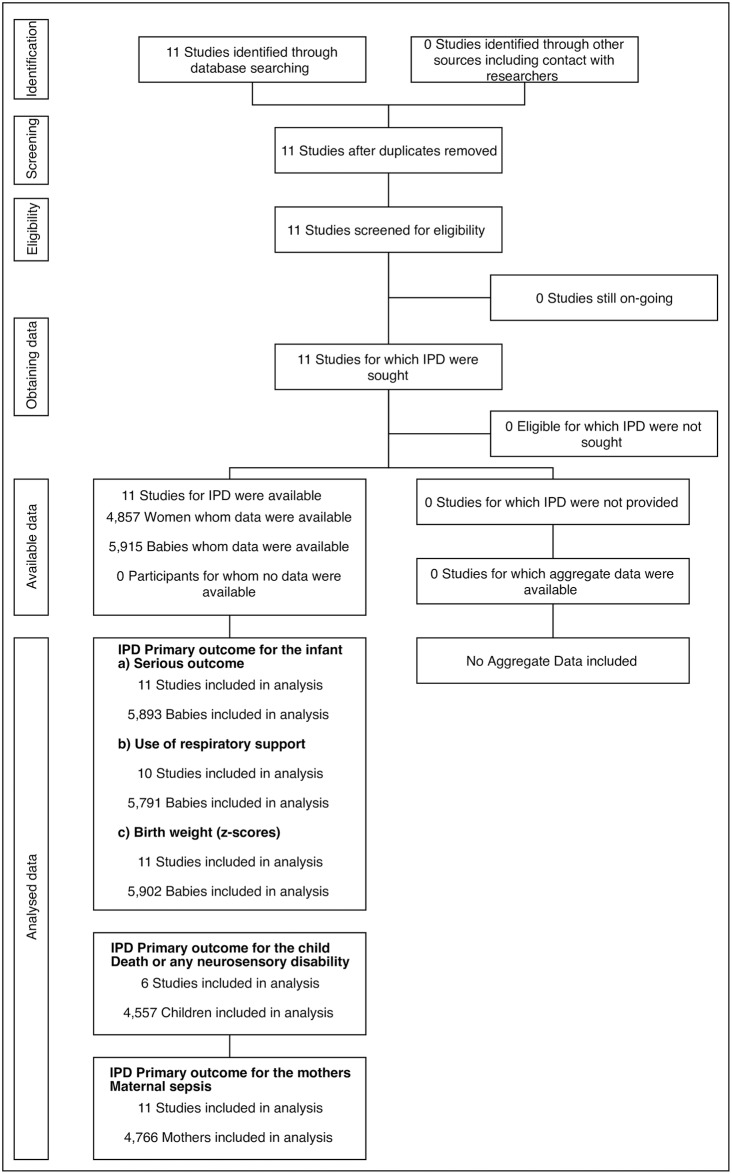
PRECISE PRISMA-IPD flow diagram. IPD, individual participant data; MA, meta-analysis; PRECISE, Prenatal Repeat Corticosteroid International IPD-MA Study: assessing the effects using the best level of Evidence; PRISMA, Preferred Items for Reporting for Systematic Review and Meta-analysis.

Eleven randomised trials were identified from the search, and each provided data for the IPD-MA [20–34], with a total of 4,857 women and 5,915 infants contributing data to the analyses (Fig 1).

Five trials were from the US [23,24,26,27,33]; two from Canada [20,28]; and one each from Australia and New Zealand [21], Finland [30], India [25], and the UK [31]. Details of the studies are described in Table 1.

**Table 1:** Included trials and their characteristics.

Trial	Participants included in IPD	Interventions	Comparator	Outcomes
Aghajafari 2002, Canada [20]	12 women (16 fetuses) 24–30 weeks’ gestation, at continued increased risk of PTB 7 or more days following a single course of ACS	2× 12 mg betamethasone (Celestone Soluspan with 6 mg betamethasone phosphate and 6 mg of betamethasone acetate) 24 hours apart repeated weekly until 33 weeks or birth, if still at risk of PTB	saline placebo, two courses, 24 hours apart	**Primary**: Perinatal or neonatal mortality or neonatal morbidity (including severe RDS, IVH [grade 3 or 4], PVL, BPD, or NEC).
				**Secondary neonatal**: Weight, length, and head circumference at birth, neonatal infection, ROP, stay in NICU, use of ventilation with intubation, and PDA needing pharmacological treatment or surgery.
				**Secondary maternal**: Chorioamnionitis and maternal infection after delivery.
Crowther 2006, Australia and New Zealand [21,22]	982 women (1,147 fetuses) at <32 weeks’ gestation who had received initial treatment with corticosteroid ≥7 days previously	1× 11.4 mg betamethasone (Celestone Chronodose with 7.8 mg betamethasone sodium phosphate and 6 mg betamethasone acetate); repeated weekly only if still at risk of PTB until <32 weeks	saline placebo	**Primary**: Frequency and severity of RDS; severity of any respiratory disease present; need for and duration of oxygen therapy; need for and duration of mechanical ventilation via an endotracheal tube (including high frequency ventilation); and weight, length, and head circumference at birth and at discharge from hospital.
				**Secondary**: Clinical chorioamnionitis requiring intrapartum antibiotics, maternal postpartum pyrexia ≥38.0 °C, any side effects of the injection for the mother, and other measures of neonatal morbidity for the infant.
Garite 2009, USA [23]	437 women (577 fetuses) at 25 to <33 weeks’ gestation who had completed a single course of corticosteroids before 30 weeks, and at least 14 days before inclusion, and were judged to have a recurring threat of PTB in the coming week	2× 12 mg betamethasone (as 6 mg betamethasone sodium phosphate and 6 mg betamethasone acetate), 24 hours apart or 4× 6 mg dexamethasone, 12 hours apart repeated once if birth expected in ≤7 days	saline placebo	**Primary**: Composite neonatal morbidity—RDS, BPD, severe IVH, PVL, proven sepsis, NEC, or perinatal death.
				**Secondary**: GA at delivery, birth weight, IUGR/SGA, head circumference, ventilator days, days requiring supplemental oxygen, need for surfactant therapy, hospital days, pneumothorax, maternal infectious morbidity (chorioamnionitis and postpartum endometritis).
Guinn 2001, USA [24]	502 women (496 fetuses) 24 to <33 weeks’ gestation who had not delivered 1 week after receipt of a single course	2× 12 mg betamethasone, 24 hours apart repeated weekly until 34 weeks	saline placebo	**Primary**: Composite neonatal morbidity (including severe RDS, BPD, severe IVH, PVL, proven sepsis, NEC, perinatal death). For the IPD-MA, some diagnostic criteria differ from original publication [24], and data analyses included all the infants.
Mazumder 2008, India [25]	76 women (76 fetuses) 25–33 weeks gestation who had not delivered within 7 days of first betamethasone course	2× 12 mg betamethasone (as betamethasone sodium phosphate), 24 hours apart, repeated weekly until 34 weeks’ gestation	no further treatment	**Primary**: Severe RDS.
				**Secondary**: Short-term neonatal morbidity (severe RDS, IVH, NEC, PDA, BPD, sepsis, ROP, deaths within 28 days), intrauterine growth (birth head circumference, length, and weight), medium-term neurodevelopmental outcomes, medium-term postnatal growth (head circumference, weight, and recumbent length at 6 months).
McEvoy 2002, USA [26]	37 women (37 fetuses) at 25–33 weeks’ gestation who remained undelivered 1 week after their first course of ACS	2× 12mg betamethasone (Celestone Soluspan with 6 mg betamethasone phosphate and 6 mg of betamethasone acetate), 24 hours apart repeated weekly until 34 weeks’ gestation or until birth	weekly saline placebo until 34 weeks’ gestation or until birth	**Primary**: Measurement of FRC.
				**Secondary**: Measurement of respiratory compliance and other important clinical respiratory outcomes including need for surfactant, days on oxygen, and mechanical ventilation.
McEvoy 2010, USA [27]	85 women (113 fetuses) at 26 to <34 weeks’ gestation who had been given an initial course of ACS and remained undelivered 14 days after therapy but still at high risk for preterm delivery	2× 12 mg betamethasone (Celestone Soluspan with 6 mg betamethasone phosphate and 6 mg of betamethasone acetate), 24 hours apart with a single repeat course of ACS	placebo	**Primary**: Measurement of passive respiratory mechanics and FRC within 72 hours of age, weekly for first month, monthly until discharge.
				**Secondary**: Clinical outcomes at discharge, follow-up pulmonary function tests at 6 months, 1 year, and 2 years corrected age; clinical respiratory outcomes, growth measurements, and neurodevelopmental outcome will be followed through 2 years of age.
Murphy 2008, Canada [28,29]	1,858 women (2,318 fetuses) at 25–32 weeks gestation who remained undelivered 14–21 days after an initial course of ACS and continued to be at high risk of PTB	2× 12 mg betamethasone (Celestone with 6 mg betamethasone sodium phosphate and 6 mg betamethasone acetate), 24 hours apart, repeated fortnightly until 33 weeks’ gestation	Placebo group—dilute concentration of aluminium monostearate	**Primary**: Perinatal or neonatal mortality or neonatal morbidity (including severe RDS, IVH (grade 3 or 4), PVL, BPD, or NEC).
				**Secondary neonatal**: Weight, length, and head circumference at birth, neonatal infection, ROP, stay in NICU, use of ventilation with intubation, and PDA needing pharmacological treatment or surgery.
				**Secondary maternal**: Chorioamnionitis and maternal infection after delivery.
Peltoniemi 2007, Finland [30]	249 women (326 fetuses) with imminent delivery before 34 weeks’ gestation who remained undelivered for >7 days after a single course of betamethasone	1× 12 mg betamethasone (Celestone Chronodose with betamethasone phosphate and betamethasone acetate) repeated once if birth expected in <48 hours	saline placebo	**Primary**: Survival without RDS or severe IVH (grade 3 or 4).
				**Secondary**: Cystic PVL, NEC of grade ≥2, BPD, PDA.
TEAMS, UK [31,32]	156 women (182 fetuses) <32 weeks gestation and had received one course of ACS	2× 12 mg betamethasone, 12 or 24 hours apart, usually repeated every 7 days but could be 10–14 days depending on unit’s protocol	placebo	**Primary**: Death in first year after birth, developmental quotient <85 at 2 years.
				**Secondary short-term**: Intrauterine death after 24 weeks gestation, neonatal death, postneonatal death, RDS, pneumothorax or other pulmonary air leak, intracerebral abnormality diagnosed on ultrasound scan before discharge, NEC, CLD, evidence of infection on blood culture before and after first 48 hours after birth, birth weight, head circumference at birth, chorioamnionitis, maternal postpartum infection.
				**Secondary long-term**: Growth at age 2 years (corrected), respiratory symptoms at age 2 years (corrected), sub-scale scores for the Vineland Adaptive Behaviour Scales at age 2 years (corrected), blood pressure at age 2 years (corrected), readmission to hospital.
Wapner 2006, USA [33,34]	495 women (594 fetuses) 23 to <32 weeks’ gestation and at risk for spontaneous PTB for any reason or placenta praevia or chronic abruption and received a full course of corticosteroids within the previous 7 days	2× 12 mg betamethasone (as 6 mg betamethasone sodium phosphate and 6 mg betamethasone acetate), 24 hours apart or 4× 6 mg dexamethasone 12 hours apart, repeated weekly until 34 weeks gestation	placebo.	**Primary**: Stillbirth or neonatal mortality, severe RDS, grade 3–4 IVH, PVL, CLD.
				**Secondary maternal**: Chorioamnionitis, postpartum endometritis, premature PROM, time from randomisation to PROM, time from randomisation to delivery, abnormal 1-hour glucose tolerance test, gestational diabetes, pulmonary oedema, infectious morbidity (highest temperature within 24 hours of birth, sepsis, need for antibiotics), days hospitalised postpartum, IL-1, IL-2, IL-4, IL-6, IL-10, IFN-γ, cortisol, rehospitalisations after delivery until 6 weeks postpartum.
				**Secondary placental**: Weight, histologic chorioamnionitis, fibrosis, vascular changes.
				**Secondary neonatal**: Birth weight, head circumference, upper arm circumference, neonatal infectious morbidity (sepsis, suspected sepsis, pneumonia), neonatal noninfectious morbidity (RDS, NEC, gastrointestinal perforation, seizures/encephalopathy, PDA, hypertension, ROP), days in NICU, GA at delivery, IL-1, IL-2, IL-4, IL-6, IL-10, IFN-γ, cortisol, TSH, free T4 and cholesterol levels, ABR interpeak interval (I–V).
				**Secondary infant**: Frequency of hospitalisations since birth, weight, and head circumference at the 24-month exam, hypertension at the 24-month exam, cerebral palsy (subclassified as mild, moderate, or severe, diagnosed at the 24-month exam), neonatal or infant death, Motor and Mental Scales score from the Bayley Scales of Infant Development II at 24 months’ corrected age, Intelligence Scale score from the McCarthy Scales of Children Abilities at 36 months’ corrected age.

**Abbreviations**: ABR, auditory brainstem response; ACS, antenatal corticosteroids; BPD, bronchopulmonary dysplasia; CLD, chronic lung disease; FRC, functional residual capacity; GA, gestational age; IFN-γ, interferon gamma; IL, interleukin; IPD, individual participant data; IUGR, intrauterine growth restriction; IVH, intraventricular haemorrhage; MA, meta-analysis; NEC, necrotising enterocolitis; NICU, neonatal intensive care unit; PDA, patent ductus arteriosus; PROM, prelabour rupture of the membranes; PTB, preterm birth; PVL, periventricular leucomalacia; RDS, respiratory distress syndrome; ROP, retinopathy of prematurity; SGA, small for gestational age; TEAMS, Trial of the Effects of Antenatal Multiple courses of Steroids versus a single course; TSH, thyroid stimulating hormone.

Individual study protocols varied in relation to the amount and number of doses used in a course of repeat treatment, the interval between courses, and the latest gestation that repeat treatment courses could be given. In most trials, a course of treatment was two doses, usually given 24 hours apart, although for two trials, a course of treatment was a single dose [21,30]. Seven of the trials allowed a course of repeat treatment at 7-day intervals [20,21,24,25,26,31,33], and one trial at 14-day intervals [28]. Most of these trials repeated the course of treatment until 33–34 weeks gestation [24,25,26,28,31,33]. In two trials [20,21], a course of repeat treatment was only given if the risk of preterm birth remained: until 32 weeks for one trial [21] and 33 weeks for the other [20]. Three trials specifically targeted women for ‘rescue therapy’ (repeat doses only given when preterm birth was again considered imminent) [23,27,30]. The characteristics of the populations, including the main reason at risk of preterm birth at trial entry, for the included trials are given in Table 2. Not all trials were able to provide data for all the endpoints analysed.

**Table 2:** Characteristics of mothers and main reasons for PTB at trial entry for the included trials.

Characteristic	Aghajafari 2002	Crowther 2006	Garite 2009	Guinn 2001	Mazumder 2008	McEvoy 2002	McEvoy 2010	Murphy 2008	Peltoniemi 2007	TEAMS [31]	Wapner 2006
Maternal age (years)	32.4 (5.7)	30.4 (6)	29 (6.2)	27.5 (7)	26.6 (4.2)	24.8 (5.9)	27.7 (7)	29.1 (6.2)	30.7 (5.8)	28.6 (6)	25.9 (5.9)
GA (weeks)	27.4 (1.9)	28.3 (2.2)	29.6 (2.1)	29 (2.7)	30.2 (1.6)	29.9 (2.4)	30.1 (2)	29.3 (2)	30.4 (2.6)	28.8 (1.9)	28.1 (2.4)
Previous pregnancies	7 (58)	671 (68)	257 (59)	338 (67)	48 (63)	15 (41)	69 (81)	1,338 (72)			435 (88)
Multiple pregnancy	4 (33)	155 (16)	141 (32)	73 (15)	7 (9)^†^	0 (0)	28 (33)	390 (21)	70 (28)	20 (12)	101 (20)
Previous PTB (<37 weeks gestation)	2 (16.7)	164 (16.7)		142 (28.3)			21 (13.3)	638 (34.3)	11 (4.4)		244 (45.9)
Main reason for risk of PTB											
Preterm labour	NA	257 (26)	NA	270 (54)	15 (20)	31 (84)	67 (79)	1,554 (84)	83 (33)	46 (28)	205 (41)
Placental abruption	NA	44 (4)	NA	35 (7)	0 (0)	1 (3)	8 (9)	NA	55 (22)	NA	25 (5)
Placenta praevia	NA	137 (14)	NA	38 (8)	15 (20)	2 (5)	8 (9)	NA	NA	3 (2)	30 (6)
Ruptured membranes	5 (42)	334 (34)	0 (0)	120 (24)	12 (16)	19 (51)	17 (20)	291 (16)	95 (38)	30 (19)	0 (0)
Antepartum haemorrhage	2 (17)	282 (29)	NA	44 (9)	0 (0)	3 (8)	4 (5)	260 (14)	NA	2 (1)	0 (0)
Pre-eclampsia	NA	97 (10)	NA	54 (11)	14 (18)	4 (11)	9 (11)	96 (5)	55 (22)	14 (9)	0 (0)
Fetal growth restriction	0 (0)	51 (5)	NA	35 (7)	3 (4)	0 (0)	7 (8)	159 (9)	64 (25)	5 (3)	0 (0)
Suspected fetal jeopardy	0 (0)	NA	NA	33 (7)	10 (13)	0 (0)	4 (5)	218 (12)	155 (62)	5 (3)	0 (0)
Cervical incompetence	4 (33)	82 (8)	NA	NA	0 (0)	5 (14)	25 (29)	909 (49)	NA	1 (1)	71 (14)
Maternal disease	3 (25)	NA	NA	152 (30)	6 (8)	2 (5)	15 (18)	391 (21)	NA	4 (2)	0 (0)
Multiple pregnancy	4 (33)	43 (4)	141 (32)	73 (15)	2 (3)	0 (0)	28 (33)	370 (20)	70 (28)	16 (10)	101 (20)

Figures are numbers (percentages) or mean and standard deviation.

^†^Only data from firstborn available for analysis.

**Abbreviations**: GA, gestational age; NA, Not Available; PTB, preterm birth; TEAMS, Trial of the Effects of Antenatal Multiple courses of Steroids versus a single course.

### Risk of bias across studies

Overall the risk of bias was low across the included studies, with some variation between the trials (Table 3).

**Table 3:** Risk of bias within studies.

Trial	Randomisation^1^	Concealment^2^	Performance^3^	Detection^4^	Attrition^5^	Reporting^6^
Aghajafari 2002	Low	Low	Low	Unclear	Low	Low
Crowther 2006	Low	Low	Low	Low	Low	Low
Garite 2009	Low	Low	Low	Low	Low	Low
Guinn 2001	Low	Low	Low	Low	Low	Low
Mazumder 2008	Low	Low	High	Unclear	Unclear	Unclear
McEvoy 2002	Low	Low	Low	Low	Low	Low
McEvoy 2010	Low	Low	Low	Low	Low	Low
Murphy 2008	Low	Low	Low	Low	Low	Low
Peltoniemi 2007	Low	Low	Low	Low	Unclear	Low
TEAMS [31] 1999	Low	Low	Low	Low	Unclear	Low
Wapner 2006	Low	Low	Low	Low	Unclear	Low

^1^Random sequence generation.

^2^Allocation concealment.

^3^Blinding of participants and personnel.

^4^Blinding of outcome assessment.

^5^Incomplete outcome data.

^6^Selective reporting.

**Abbreviations**: TEAMS, Trial of the Effects of Antenatal Multiple courses of Steroids versus a single course.

### Primary outcomes for the infant

#### Serious outcome

**Fig 2:**
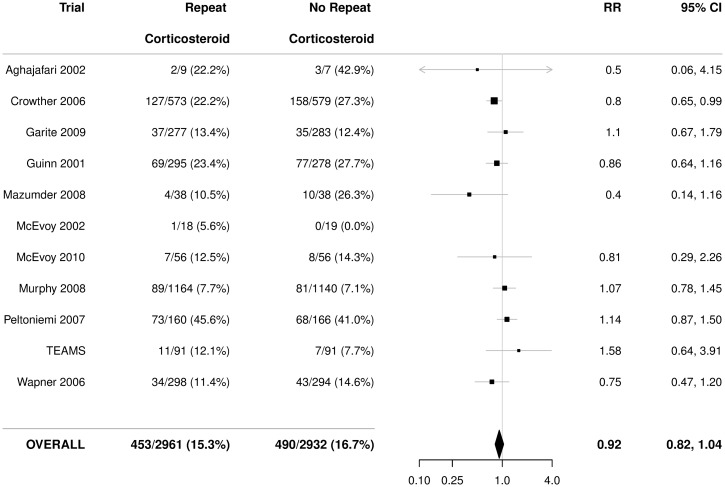
Primary outcome for the infant: Serious outcome. Serious outcome is defined as any death (fetal, neonatal, infant, or child), severe respiratory disease as defined by the trialists, grade 3 or 4 IVH, CLD (oxygen dependent at 36 weeks postmenstrual age), definite NEC, stage 3 or worse ROP in the better eye, or cystic PVL. Figures are numbers (percentages) with RR and 95% CI as treatment effect. *p*-value for heterogeneity = 0.33. CI, confidence interval; CLD, chronic lung disease; IVH, intraventricular haemorrhage; NEC, necrotising enterocolitis; PVL, periventricular leucomalacia; ROP, retinopathy of prematurity; RR, relative risk; TEAMS, Trial of the Effects of Antenatal Multiple courses of Steroids versus a single course.

There was no statistically significant effect of repeat prenatal corticosteroids compared with no repeat treatment on the risk of serious outcome for the infant (RR 0.92, 95% CI 0.82 to 1.04), with no significant heterogeneity (*p* = 0.33) (Fig 2).

#### Use of respiratory support

**Fig 3:**
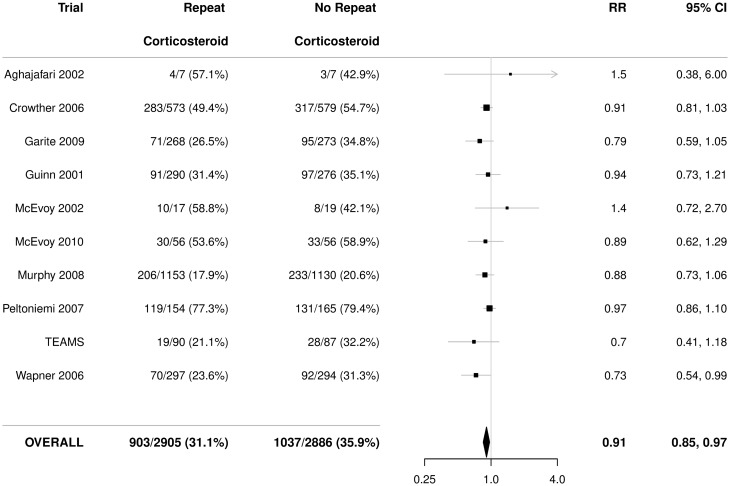
Primary outcome for the infant: Use of respiratory support. Figures are numbers (percentages) with RR and 95% CI as treatment effect. *p*-value for heterogeneity = 0.64. CI, confidence interval; RR, relative risk; TEAMS, Trial of the Effects of Antenatal Multiple courses of Steroids versus a single course.

There was a statistically significant reduction in the use of respiratory support in infants exposed to repeat prenatal corticosteroids compared with infants not exposed (RR 0.91, 95% CI 0.85 to 0.97), with no significant heterogeneity (*p* = 0.64) (Fig 3). The NNT to benefit was 21 women/fetuses (95% CI 14 to 41) to prevent one infant from needing respiratory support.

#### Birth weight

**Fig 4:**
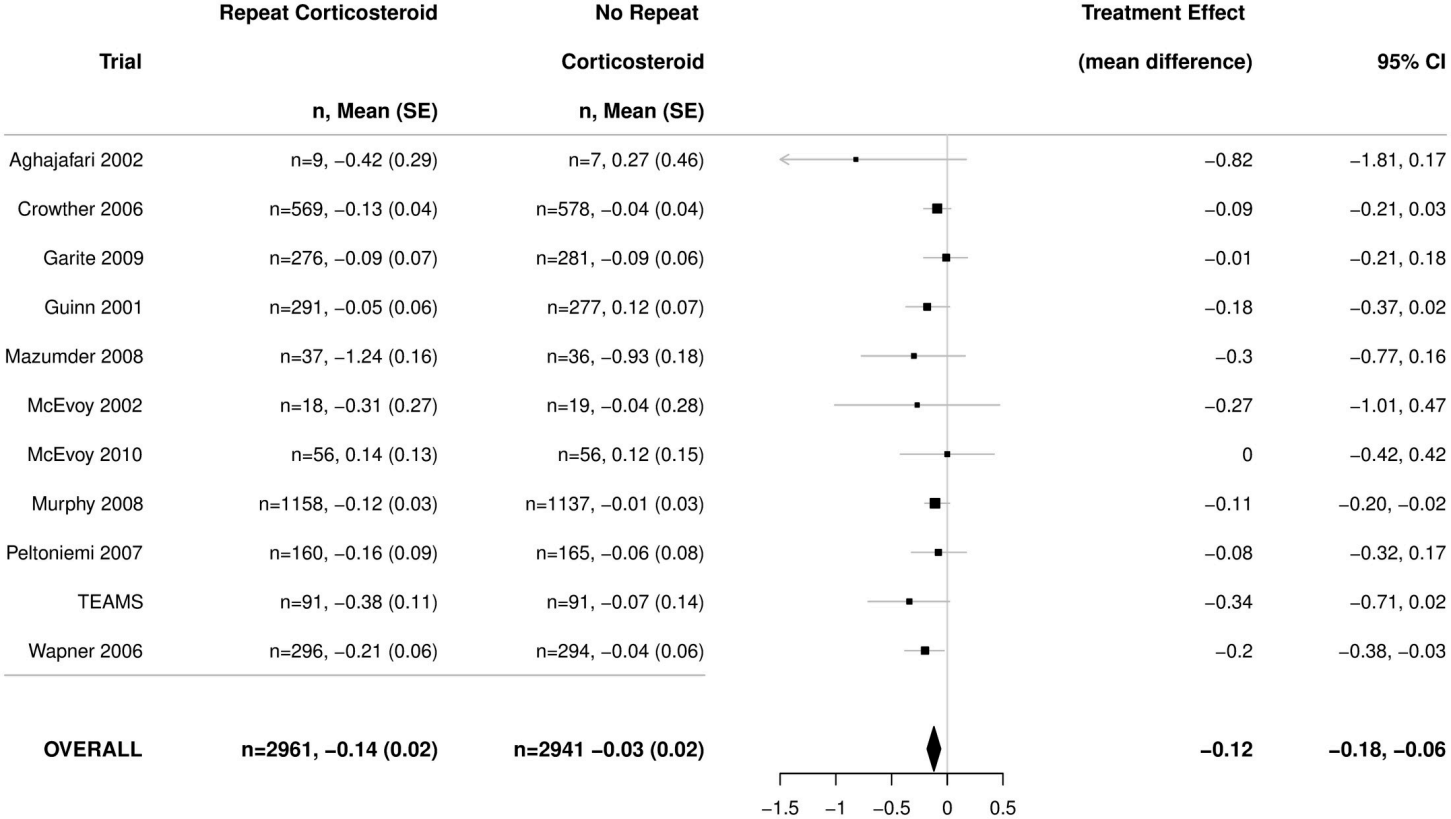
Primary outcome for the infant: Birth weight (z-scores). Figures are mean and standard deviation, with adjusted mean difference as treatment effect and 95% CI. *p*-value for heterogeneity = 0.80. CI, confidence interval; TEAMS, Trial of the Effects of Antenatal Multiple courses of Steroids versus a single course.

There was a significantly lower birth weight z-score in infants exposed to repeat prenatal corticosteroids compared with unexposed infants (mean difference ‒0.12, 95% CI ‒0.18 to ‒0.06) (Fig 4), with no heterogeneity among the trials (*p* = 0.80).

#### Primary outcomes for the child

**Fig 5:**
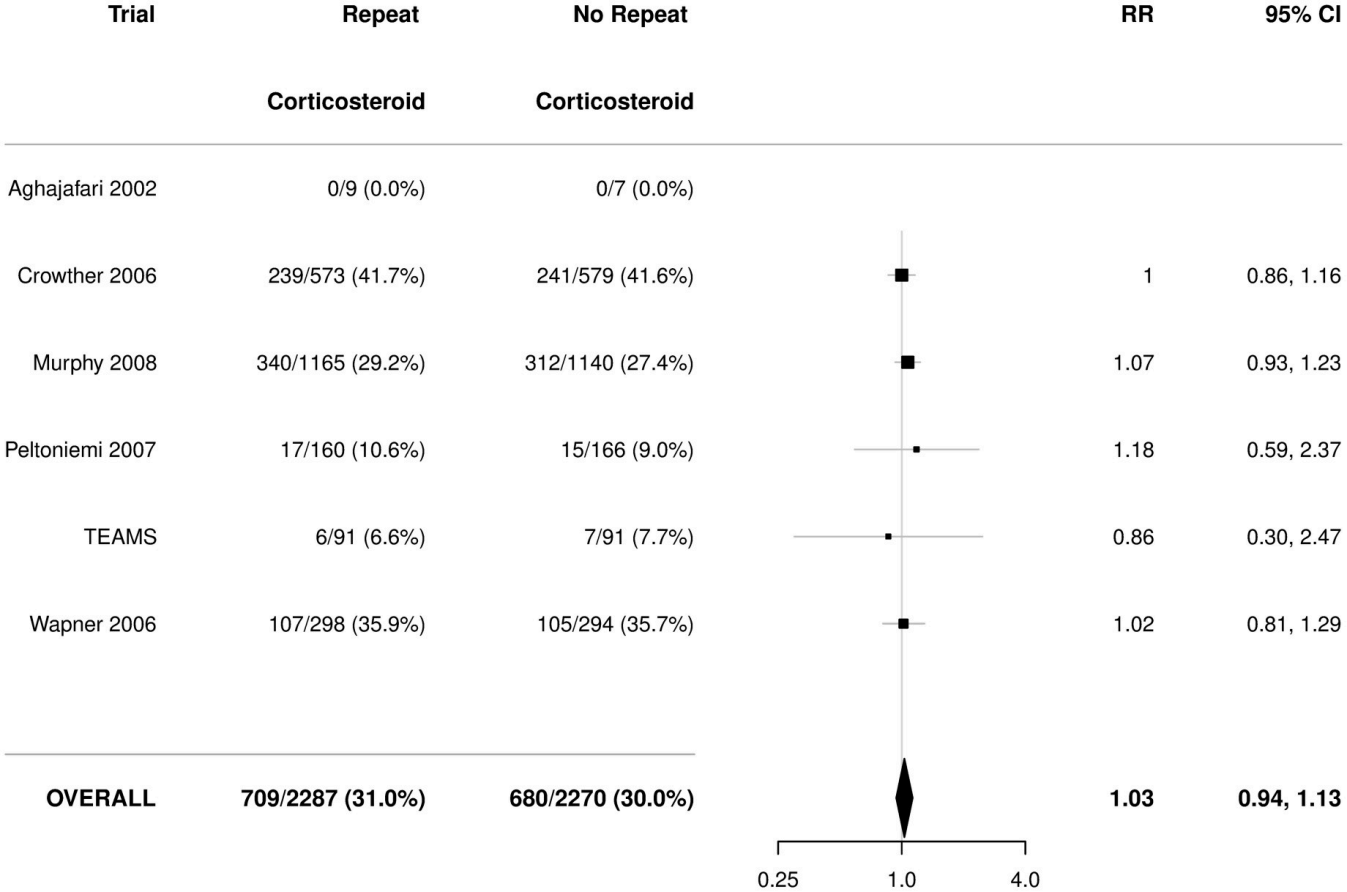
Primary outcome for the child: Death or any neurosensory disability. Neurosensory disability at childhood follow-up defined as developmental delay or intellectual impairment (developmental quotient or intelligence quotient more than one standard deviation below the mean), cerebral palsy (abnormality of tone with motor dysfunction), blindness (corrected visual acuity worse than 6/60 in the better eye), or deafness (hearing loss requiring amplification or worse). Figures are numbers (percentages) with RR and 95% CI. *p*-value for heterogeneity 0.96. CI, confidence interval; RR, relative risk; TEAMS, Trial of the Effects of Antenatal Multiple courses of Steroids versus a single course.

There was no statistically significant effect of treatment with repeat prenatal corticosteroids on the risk of death or neurosensory disability for the child (Fig 5).

#### Primary outcomes for the woman

**Fig 6:**
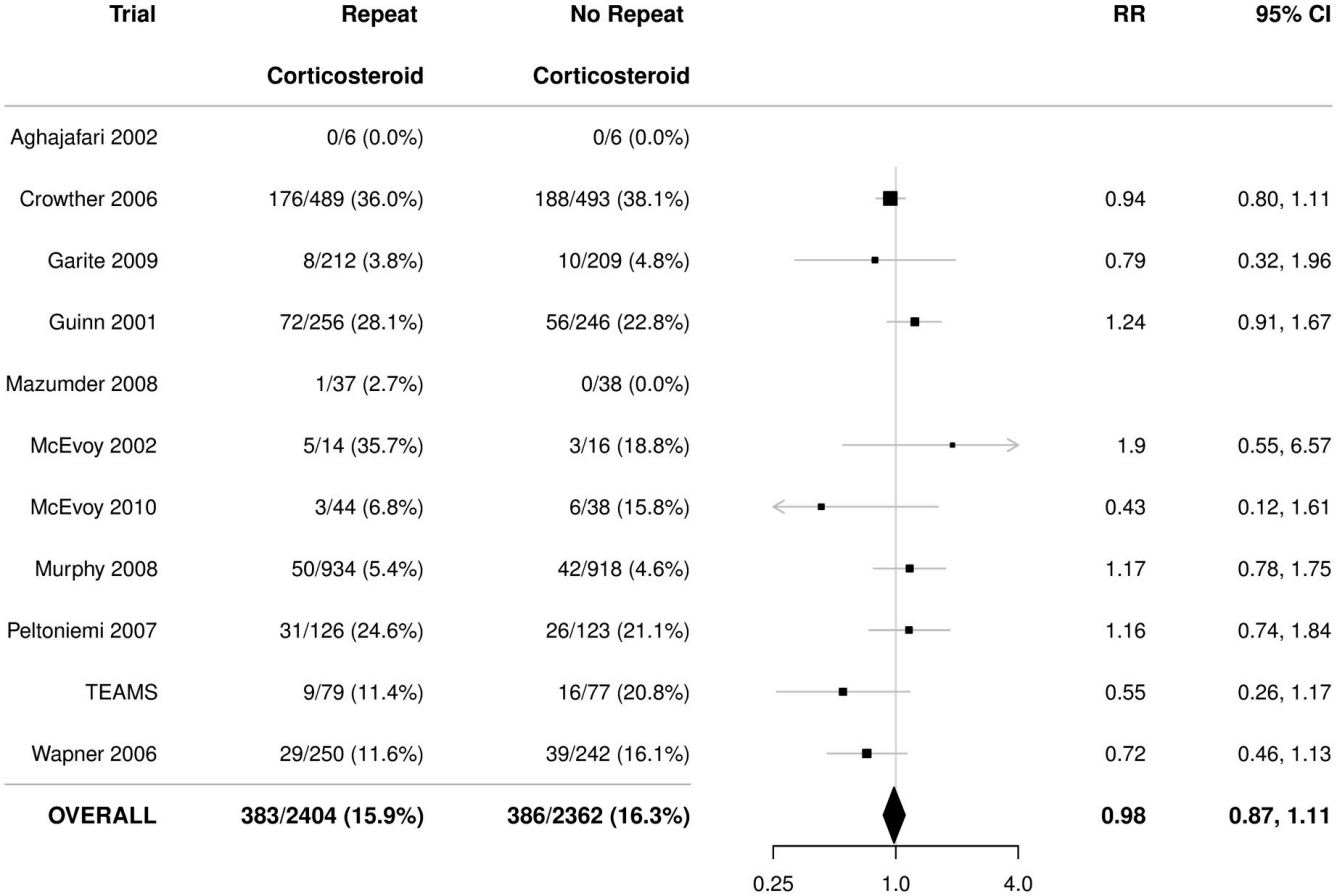
Primary outcomes for the women: Maternal sepsis. Maternal sepsis defined as one of the following: chorioamnionitis during labour, pyrexia after trial entry requiring the use of antibiotics, puerperal sepsis, intrapartum fever requiring the use of antibiotics, or postnatal pyrexia. Figures are numbers (percentages) with RR and 95% CI. *p*-value for heterogeneity 0.22. CI, confidence interval; RR, relative risk; TEAMS, Trial of the Effects of Antenatal Multiple courses of Steroids versus a single course.

For the primary maternal outcomes of maternal sepsis, no statistically significant effect was seen with repeat corticosteroids using the data available for the IPD (Fig 6).

### Secondary outcomes

#### For the infant

No statistically significant differences were seen for many of the secondary infant outcomes (S1 Table). Fewer infants in the repeat corticosteroid group compared with infants in the no-repeat group required resuscitation at birth, ongoing respiratory support, surfactant treatment, or oxygen supplementation, they were less likely to have respiratory distress syndrome, and any respiratory disease was less severe (S1 Table). However, birth weight, length, and head circumference measurements were all lower in the repeat corticosteroid group, and there were proportionately more small for gestational age infants compared with the no-repeat group (S1 Table). Systolic, diastolic, and mean arterial blood pressures were significantly lower in infants exposed to repeat corticosteroids compared with nonexposed infants, although fewer than 10% of infants had data reported for these outcomes (S1 Table).

#### For the children

For the children, no statistically significant differences were seen for the outcomes of death, neurosensory impairments, any neurosensory disability, major neurosensory disability, child behaviour, or respiratory disease at follow-up; however, weight z-scores and systolic blood pressure were lower (S2 Table).

#### For the woman

No statistically significant differences were seen in any of the secondary outcomes for the woman, including the individual components of the primary outcome of maternal sepsis, preterm prelabour rupture of the membranes, hypertension, mode of birth, postpartum haemorrhage, breast feeding at discharge, postnatal depression, or adverse effects of corticosteroid therapy (including gastrointestinal upset, glucose intolerance, insomnia, pain at the injection site, bruising at the injection site, infection at injection site, weight gain, Cushingoid appearance). For the composite outcome of adverse effects of corticosteroid therapy, the *p*-value for heterogeneity was <0.0001, indicating differences between the studies not accounted for by random variation, such as differences in the placebo used (S3 Table). There were no maternal deaths reported.

### Subgroup analyses

When sufficient data existed, subgroup comparisons were conducted using the five primary outcomes for the infant, child, and mother, as well as the individual components of each composite outcome. Not all planned subgroup analyses were possible because of unavailability of data. When subgroup analyses were possible, not all trials had data to contribute.

The prespecified participant level characteristics that were analysed were the reason the woman was considered to be at risk of preterm birth, the number of fetuses in utero, gestational age when first trial treatment course was given (trial entry), the time prior to birth that the last dose of trial treatment course was given, the number of courses received, the interval between courses, and the total dose received.

#### Reason the woman was considered to be at risk of preterm birth

There were few clear differences in treatment effects among subgroups of reasons the women were considered at risk of preterm birth. Reductions in birth weight, head circumference, and length due to receiving repeat corticosteroids were greater when the reason for being at risk of preterm birth was preterm labour than when it was not (S4 Table). For those whose reason for preterm birth was fetal growth restriction, reduction in birth weight due to repeat corticosteroid treatment was less likely than when the reason for preterm birth was not fetal growth restriction (S4 Table). Developmental delay/intellectual impairment was more likely when the reason for preterm birth was maternal disease than when it was not (S4 Table).

#### Number of fetuses in utero (singleton versus multiple birth)

There were no significant differences in treatment effects among the subgroups by the number of fetuses in utero (S5 Table).

#### Gestational age when first repeat treatment course was given

Reductions in birth weight, head circumference, and length were significantly greater for infants whose mothers had received corticosteroids at an earlier gestational age. Women starting treatment at earlier gestations were a subgroup who also received higher total exposure overall (S6 Table).

#### Time prior to birth last dose of trial treatment course was given

There were no significant differences in treatment effects for any of the major outcomes according to categories of time prior to last dose of treatment until birth (S7 Table).

#### Number of courses of repeat prenatal corticosteroids received

The number of courses of repeat prenatal corticosteroids received varied by trial due to the different treatment protocols. The number of courses also varied by participant characteristics such as the gestational age at which study treatment commenced, with women commencing at an earlier gestational age having more time to receive more courses of treatment provided birth did not occur. Half of the children in the analyses were exposed to only one course of treatment. Only 3% of children were exposed to six or more courses of treatment. CIs for this subgroup were therefore wide because of the smaller number of participants in the analysis. Because the number of courses received was a postrandomisation measurement, these analyses were considered exploratory, although still clinically important, comparisons. For the primary outcomes of serious outcome and use of respiratory support for the infant, treatment effects differed according to the number of courses of repeat treatments received. Benefits of repeat corticosteroid were observed for infants who were exposed to between 2 to 5 courses of repeat corticosteroids compared with infants exposed to only a single repeat course or 6 or more repeat courses. There were greater reductions in birth weight and length z-scores for infants exposed to 4 or more repeat courses and in head circumference for infants exposed to two or more repeat courses (S8 Table).

#### Minimum actual interval between repeat treatment courses

The minimum interval between repeat treatment courses varied by trial because of the different treatment protocols and different participants. Intervals between repeat courses of 1–7 days and 8 or more days showed a reduction in serious outcome and the use of respiratory support for infants exposed to repeat courses compared with a single repeat course. For the other outcomes, apart from death at any time, no obvious differences were seen between groups for different minimum interval between repeat treatment courses (S9 Table).

#### Actual total dose of trial treatment received

The dose of trial treatment received varied by trial because of the different trial protocols and also because of the number of courses of treatment actually received. Differences in treatment effects were seen for serious outcome for the infant, use of respiratory support, death at any time, and head circumference depending on the total dose received. Benefits of repeat corticosteroids were seen for serious outcome for the infant and for use of respiratory support with larger doses (>24 mg) and for death at any time with total doses between >24 mg to 48 mg of repeat corticosteroid compared with total doses received of ≤24 mg (S10 Table). Head circumference z-scores at birth were reduced if total doses of corticosteroid received was >24 mg compared with total doses of ≤24 mg (S10 Table).

## Discussion

### Summary of evidence

The major findings from the PRECISE IPD-MA are that for women at risk of preterm birth, use of repeat prenatal corticosteroids following an initial course 7 or more days previously does not reduce the risk of serious outcome for their infant but does reduce the risk of their infant needing respiratory support after birth. The number of women who need to be treated to prevent one infant needing respiratory support is 21 (95% CI 14 to 41). This benefit is associated with their infant being less likely to have respiratory distress syndrome, having less severe respiratory disease, and being less likely to require resuscitation at birth, surfactant treatment, and oxygen supplementation. These respiratory benefits are not at the expense of any short-term complications for the mother. However, z-scores for weight are significantly lower in infants whose mothers receive repeat prenatal corticosteroids (mean difference 80 g), as are z-scores for head circumference and length at birth and the proportion of infants born small for gestational age. The clinical significance of the reduction in birth weight z-scores is unclear. There appear to be no longer-term clinical benefits or harms of repeat prenatal corticosteroids in childhood, as assessed by neurosensory disabilities, respiratory outcomes, or behaviour, and few differences in body size, although weight z-scores are lower by ‒0.11 SD (95% CI ‒0.19 to ‒0.03). The clinical importance of this reduction in weight z-score at early childhood follow-up, in the absence of clear evidence of effects on head size, length/height, or neurosensory outcomes is unclear. There is also a small reduction in systolic blood pressure of uncertain clinical importance. Ex-preterm survivors have higher blood pressure in late adolescence or early adulthood than do term controls [35], which may predispose them to more cardiovascular disease in adult life. Since blood pressure tracks through childhood into late adolescence in preterm survivors [36], a small reduction in child blood pressure may be a long-term benefit of repeat doses of corticosteroids. Follow-up into adolescence and adult life is needed to clarify this.

The results from this IPD-MA are consistent with the aggregate meta-analysis in the Cochrane review assessing the use of repeat prenatal corticosteroids in women who remain at risk of preterm birth after an initial course of corticosteroids [9]. The new information that this IPD-MA has clarified is that no specific subgroup of infants benefitted more or less from exposure to repeat prenatal corticosteroids, including plurality, the gestational ages when repeat treatment was first given, or the time prior to birth the last repeat treatment was given.

In three subgroup analyses involving postrandomisation events (the minimum actual interval between repeat treatment courses, the number of repeat prenatal corticosteroids courses received, and the total dose of corticosteroids received), differences were seen between subgroups. The risk of a serious outcome for the infant and the need for respiratory support were reduced when exposed to at least 2 repeat treatment courses and to total doses of more than 24 mg of repeat treatment. Death at any time was reduced with exposure to total doses of between >24 mg to 48 mg.

The number of repeat prenatal corticosteroid treatment courses and the total dose of repeat prenatal corticosteroids also affected body size z-scores at birth, with exposure to increasing numbers of repeat treatment courses or larger total doses associated with greater reductions in growth. There were greater reductions in birth weight for infants exposed to 4 or more repeat courses. To provide clinical benefit with the least harm in women who remain at high risk of preterm birth 7 or more days after an initial course of corticosteroids, it would seem prudent to limit the use of any repeat corticosteroids to a total dose of between >24 mg to 48 mg and a maximum of 3 repeat courses of treatment.

### Strengths and limitations

IPD-MA is recognised as the gold standard for systematic reviews [11]. One of the strengths of this IPD-MA is that all the trialists were willing to be part of the PRECISE Group collaborative initiative and provide their data. All the trials used betamethasone—there are no IPD-MA data for dexamethasone.

In this way, we were able to include IPD from all the known completed randomised trials of repeat prenatal corticosteroid treatment given to women at risk of preterm birth to benefit their infants. The IPD-MA findings provide the best available evidence for decision makers at this time.

We have recently updated the search for trials (date of search 22 January 2019). No additional randomised trials of repeat prenatal corticosteroid treatment given 7 or more days after an initial course of prenatal corticosteroids were identified. Two of the trials have reported health outcomes in later childhood, one at 5 years [37] and the other 6–8 years of age [38]. Both trials found no differences between children exposed to repeat prenatal corticosteroids and those not in the risk of death or neurodevelopmental disability or in body size measurements [37,38].

Not all trials collected or were able to provide data for all of the prespecified subgroups. This limited the power to be able to detect overall or subgroup differences because event rates are low for some important maternal, infant, and childhood clinical outcomes. Neonatal hypoglycaemia was not reported in any of the trials but has recently been linked with use of antenatal corticosteroids in the late preterm period [39], so it should be assessed in any future primary studies. Several of the planned subgroup analyses were for events that happened after randomisation, including number of repeat treatments given, the timing prior to birth repeat treatment was given, and the total dose received. Interpretation of these results should be cautious because postrandomisation events may be affected by the treatment given. Although such events are more subject to bias, our purpose was to identify mothers and infants within subgroups who would benefit most from treatment. This would enable use of repeat prenatal corticosteroid treatment to be optimally targeted.

### Conclusions

The PRECISE IPD-MA found that repeat prenatal corticosteroids given to women at ongoing risk of preterm birth after an initial course reduces the likelihood of their infant needing respiratory support after birth and leads to other respiratory benefits. Body size measurements at birth are lower in infants whose mothers had received repeat prenatal corticosteroids, and the reductions in weight z-scores and systolic blood pressure seen at childhood follow-up are of uncertain clinical importance. No longer-term clinical benefits or harms are seen in childhood for neurosensory disabilities, respiratory outcomes, or behaviour.

Prenatal corticosteroids are easy to administer, relatively inexpensive, and are effective at reducing the need for respiratory support that preterm infants often require. Recommendations to use repeat prenatal corticosteroids when indicated are now included within the WHO recommendations on interventions to improve preterm birth outcomes [40] and several national clinical practice guidelines [10,41,42]. More widespread adoption could lead to significant global health and economic benefits [43].

It is reassuring that the respiratory benefit is seen among varying reasons for risk of preterm birth, in singleton and multiple pregnancies, and across a range of preterm gestational ages when repeat treatment courses are first given and times prior to birth the last repeat treatment is given. Because the benefits of reduced serious outcome and need for respiratory support for the infant are accompanied by a reduction in measures of body size at birth that are related to the number of repeat treatment courses and total dose of repeat prenatal corticosteroid treatment given, it would seem prudent to limit the use of repeat corticosteroids in women who remain at high risk of preterm birth to a total dose of between >24 mg to 48 mg and a maximum of three repeat courses of treatment.

## Supporting information

S1 Table Secondary outcomes for the infants. (DOCX)

S2 Table Secondary outcomes for the children. (DOCX)

S3 Table Secondary outcomes for the women. (DOCX)

S4 Table Subgroup analysis of treatment effects by reason the woman was considered to be at risk of PTB. PTB, preterm birth.
(DOCX)

S5 Table Subgroup analysis of treatment effects among the subgroups: Singleton versus multiple birth. (DOCX)

S6 Table Subgroup analysis of treatment effects among the subgroups of GA (weeks) when first repeat treatment course was given. GA, gestational age.
(DOCX)

S7 Table Subgroup analysis of treatment effects among the subgroups according to time (days) prior to birth last dose of trial treatment course was given. (DOCX)

S8 Table Subgroup analysis of treatment effects among the subgroups considered by number of repeat courses of treatment received. (DOCX)

S9 Table Subgroup analysis of treatment effects among the subgroups according to minimum actual interval (days) between repeat trial treatment courses. (DOCX)

S10 Table Subgroup analysis of treatment effects among the subgroups according to dose of corticosteroids received (mg). (DOCX)

S1 Text PRISMA checklist. PRISMA, Preferred Items for Reporting for Systematic Review and Meta-analysis.
(DOCX)

S2 Text PRECISE IPD-MA. Repeat prenatal corticosteroid prior to preterm birth: a systematic review and IPD-MA for the PRECISE study group Study Protocol. IPD, individual participant data; MA, meta-analysis; PRECISE, Prenatal Repeat Corticosteroid International IPD-MA Study: assessing the effects using the best level of Evidence.
(PDF)

## Abbreviations

ABR: auditory brainstem response
ACS: antenatal corticosteroids
ARR: absolute risk reduction
BPD: bronchopulmonary dysplasia
CENTRAL: Cochrane Central Register of Controlled Trials
CI: confidence interval
FRC: functional residual capacity
GEE: generalised estimating equations
IFN-γ: interferon gamma
IL: interleukin
IPD: individual participant data
IUGR: intrauterine growth restriction
MA: meta-analysis
NA: Not Available
NICU: neonatal intensive care unit
NNT: number needed to treat
PDA: patent ductus arteriosus
PRECISE: Prenatal Repeat Corticosteroid International IPD-MA Study: assessing the effects using the best level of Evidence
PRISMA: Preferred Items for Reporting for Systematic Review and Meta-analysis
PTB: preterm birth
PVL: periventricular leucomalacia
RR: relative risk
TEAMS: Trial of the Effects of Antenatal Multiple courses of Steroids versus a single course
TSH: thyroid stimulating hormone
