# Supplementary material for: Effects of repeat prenatal corticosteroids given to women at risk of preterm birth: An individual participant data meta-analysis
Source: PLoS Med. 2019 Apr 12;16(4):e1002771. doi: 10.1371/journal.pmed.1002771 (PMC6461224; doi:10.1371/journal.pmed.1002771)
Supplement: S1 Table — (DOCX) [file pmed.1002771.s001.docx]

**S2 Table. Secondary outcomes for the infants***

| Outcome | N trials | Repeat Corticosteroid | No Repeat Corticosteroid | Treatment Effect | 95%CI | P value** |
| --- | --- | --- | --- | --- | --- | --- |
| Fetal, neonatal or later death (up to primary hospital discharge) | 11 | 106/2895 (3.7%) | 109/2868 (3.8%) | 0.94 | 0.72, 1.24 | 0.80 |
| Severe respiratory disease | 8 | 285/2510 (11.4%) | 346/2480 (14.0%) | 0.85 | 0.73, 0.98 | <0.001 |
| Severe IVH (grade 3 and 4) | 11 | 31/2066 (1.5%) | 33/2034 (1.6%) | 0.95 | 0.57, 1.56 | 0.28 |
| Chronic lung disease | 10 | 203/2793 (7.3%) | 200/2778 (7.2%) | 1.01 | 0.83, 1.23 | 0.77 |
| Necrotising enterocolitis | 11 | 66/2922 (2.3%) | 71/2904 (2.4%) | 0.94 | 0.67, 1.31 | 0.48 |
| Severe retinopathy of prematurity requiring treatment (Grade 3 and 4) | 8 | 28/1735 (1.6%) | 19/1720 (1.1%) | 1.34 | 0.73, 2.47 | 0.79 |
| Cystic PVL | 10 | 24/2222 (1.1%) | 31/2185 (1.4%) | 0.77 | 0.45, 1.33 | 0.89 |
| Respiratory distress syndrome | 11 | 601/2952 (20.4%) | 724/2928 (24.7%) | 0.85 | 0.77, 0.93 | 0.55 |
| IVH (any) | 11 | 231/2350 (9.8%) | 235/2322 (10.1%) | 1.00 | 0.83, 1.20 | 0.53 |
| Patent ductus arteriosus | 10 | 173/2573 (6.7%) | 217/2540 (8.5%) | 0.83 | 0.67, 1.02 | 0.05 |
| Neonatal encephalopathy | 1 | 2/36 (5.6%) | 4/37 (10.8%) | 0.51 | 0.10, 2.63 | N/A |
| Serious outcome (as defined by individual trialists) | 10 | 576/2882 (20.0%) | 626/2846 (22.0%) | 0.94 | 0.84, 1.04 | **0.05** |
| Gestational age at birth (weeks)# | 11 | n=2982, 33.4 (0.07) | n=2953, 33.6 (0.07) | -0.20 | -0.40, 0.00 | 0.59 |
| Interval between trial entry and birth (days)# | 9 | n=2844, 31.2 (0.5) | n=2812, 32.0 (0.5) | -1.19 | -2.71, 0.34 | 0.23 |
| Small for gestational age | 11 | 394/2961 (13.3%) | 325/2941 (11.1%) | 1.21 | 1.05, 1.39 | 0.74 |
| Birth weight (raw values) (g)# | 11 | n=2970, 2012 (14.7) | n=2945, 2087 (14.9) | -80 | -124, -36 | 0.67 |
| Birth weight (z-scores) # | 11 | n=2961, -0.14 (0.02) | n=2941, -0.03 (0.02) | -0.12 | -0.18, -0.06 | 0.80 |
| Head circumference at birth (raw values) (cm)# | 11 | n=2827, 30.2 (0.1) | n=2796, 30.5 (0.1) | -0.35 | -0.54, -0.16 | 0.67 |
| Head circumference at birth (z-scores)# | 11 | n=2809, -0.14 (0.02) | n=2780, 0.01 (0.02) | -0.15 | -0.22, -0.09 | 0.63 |
| Length at birth (raw values) (cm)# | 9 | n=2449, 43.1 (0.1) | n=2430, 43.7 (0.1) | -0.62 | -0.94, -0.30 | 0.75 |
| Length at birth (z-scores)# | 9 | n=2427, -0.20 (0.02) | n=2421, -0.07 (0.02) | -0.13 | -0.20, -0.06 | 0.96 |
| Apgar score less than 7 at 5 minutes | 10 | 178/2805 (6.4%) | 206/2777 (7.4%) | 0.87 | 0.71, 1.06 | 0.82 |
| Resuscitation at birth | 6 | 1012/2093 (48.4%) | 1090/2061 (52.9%) | 0.93 | 0.88, 0.99 | 0.77 |
| Duration of respiratory support (days)§ | 4 | n=246, 67 (54, 82) | n=272, 57 (48, 68) | 1.12 | 0.84, 1.49 | 0.49 |
| Use of oxygen supplementation | 9 | 1172/2601 (45.1%) | 1272/2574 (49.4%) | 0.93 | 0.88, 0.99 | 0.34 |
| Duration of oxygen supplementation (days)§ | 6 | n=705, 81 (70, 94) | n=773, 74 (65, 85) | 1.09 | 0.89, 1.34 | 0.96 |
| Use of surfactant | 10 | 552/2915 (18.9%) | 687/2890 (23.8%) | 0.84 | 0.75, 0.93 | 0.05 |
| Air leak syndrome | 9 | 54/2602 (2.1%) | 72/2590 (2.8%) | 0.75 | 0.53, 1.06 | 0.52 |
| Use of inotropic support | 5 | 151/675 (22.4%) | 200/702 (28.5%) | 0.86 | 0.72, 1.03 | 0.52 |
| Use of nitric oxide for respiratory support | 5 | 12/573 (2.1%) | 21/579 (3.6%) | 0.57 | 0.28, 1.16 | N/A |
| Early neonatal infection | 10 | 476/2547 (18.7%) | 491/2502 (19.6%) | 0.97 | 0.87, 1.08 | 0.61 |
| Proven neonatal infection in ICU | 9 | 278/2672 (10.4%) | 271/2635 (10.3%) | 1.03 | 0.87, 1.21 | 0.71 |
| Use of postnatal corticosteroids | 10 | 102/2325 (4.4%) | 86/2311 (3.7%) | 1.21 | 0.90, 1.62 | 0.37 |
| Neonatal blood pressure (systolic) (mmHg) # | 2 | n=274, 62.9 (0.68) | n=266, 64.9 (0.76) | -2.31 | -4.42, -0.20 | 0.02 |
| Neonatal blood pressure (diastolic) (mmHg)# | 2 | n=274, 35.7 (0.61) | n=267, 37.8 (0.58) | -2.19 | -3.89, -0.49 | <0.001 |
| Neonatal blood pressure (mean arterial) (mmHg) # | 2 | n=274, 44.8 (0.59) | n= 266, 46.8 (0.57) | -2.19 | -3.85, -0.53 | 0.001 |
| Weight at discharge (Z-scores)# | 3 | n=821, -0.68 (0.04) | n=815, -0.57 (0.04) | -0.12 | -0.23, -0.01 | 0.33 |
| Head circumference at discharge (Z-score)# | 3 | n=746, 0.06 (0.05) | n=723, 0.13 (0.04) | -0.06 | -0.19, 0.07 | 0.55 |
| Length at discharge (Z-score)# | 3 | n=694, -0.56 (0.06) | n=682, -0.55 (0.06) | -0.04 | -0.20, 0.12 | 0.28 |

Figures are numbers (percentages) with relative risk (RR) and 95% confidence interval (CI) as treatment effect; or # mean and standard deviation, with adjusted mean difference as treatment effect and 95% confidence interval; or § geometric mean and 95% CI.

N/A not applicable.

** Heterogeneity p values
