# Supplementary material for: Effects of repeat prenatal corticosteroids given to women at risk of preterm birth: An individual participant data meta-analysis
Source: PLoS Med. 2019 Apr 12;16(4):e1002771. doi: 10.1371/journal.pmed.1002771 (PMC6461224; doi:10.1371/journal.pmed.1002771)
Supplement: S2 Table — (DOCX) [file pmed.1002771.s002.docx]

**S3 Table. Secondary outcomes for the children**

| Outcome | N Trials | Repeat  Corticosteroid | No Repeat Corticosteroid | Treatment Effect | 95%CI | P value* |
| --- | --- | --- | --- | --- | --- | --- |
| Death (fetal, neonatal or later death up to the time of follow up) | 11 | 119/2896 (4.1%) | 120/2868 (4.2%) | 0.96 | 0.74, 1.24 | 0.75 |
|  | Neurosensory impairments | | | | | |
| Cerebral palsy | 5 | 55/1961 (2.8%) | 53/1950 (2.7%) | 1.02 | 0.70, 1.49 | 0.53 |
| Developmental delay or intellectual impairment | 4 | 532/1833 (29.0%) | 522/1819 (28.7%) | 1.02 | 0.92, 1.14 | 0.90 |
| Blindness | 4 | 24/1537 (1.6%) | 20/1508 (1.3%) | 1.14 | 0.62, 2.12 | 0.35 |
| Deafness | 4 | 24/1534 (1.6%) | 24/1507 (1.6%) | 1.02 | 0.57, 1.85 | 0.83 |
| Gross motor dysfunction** | 4 | 95/1879 (5.1%) | 95/1848 (5.1%) | 0.98 | 0.74, 1.29 | 0.97 |
| Motor delay | 4 | 380/1783 (21.3%) | 362/1776 (20.4%) | 1.05 | 0.92, 1.21 | 0.52 |
| Any neurosensory disability*** | 5 | 605/2065 (29.3%) | 585/2060 (28.4%) | 1.03 | 0.93, 1.14 | 0.91 |
| Major neurosensory disability† | 5 | 212/2006 (10.6%) | 204/1996 (10.2%) | 1.03 | 0.85, 1.25 | 0.23 |
| Weight at follow up (z-scores)# | 4 | n=1878, 0.22 (0.03) | n=1856, 0.34 (0.03) | -0.11 | -0.19, -0.03 | 0.24 |
| Head circumference at follow up (z-score)# | 4 | n=1844, 0.60 (0.03) | n=1813, 0.68 (0.03) | -0.07 | -0.15, 0.02 | 0.44 |
| Height at follow up (Z-score)# | 4 | n=1717, -0.06 (0.03) | n=1693, 0.03 (0.03) | -0.08 | -0.18, 0.01 | 0.27 |
| Child behaviour | 2 | 183/712 (25.7%) | 166/720 (23.1%) | 1.11 | 0.92, 1.35 | 0.46 |
| Respiratory disease | 4 | 426/1949 (21.9%) | 450/1941 (23.2%) | 0.96 | 0.86, 1.08 | 0.19 |
| Follow up blood pressure (systolic) (mmHg)# | 2 | n=544, 91.5 (0.56) | n=557, 93.0 (0.57) | -1.73 | -3.39, -0.08 | 0.18 |
| Follow up blood pressure (diastolic) (mmHg)# | 2 | n=522 56.7 (0.44) | n=544, 57.0 (0.47) | -0.48 | -1.81, 0.84 | 0.35 |
| Follow up blood pressure (mean arterial) (mmHg)# | 2 | n=518, 68.4 (0.45) | n=538, 69.0 (0.46) | -0.80 | -2.13, 0.53 | 0.19 |

Figures are numbers (percentages) with relative risk (RR) and 95% confidence interval (CI) as treatment effect; or # mean and standard deviation, with adjusted mean difference as treatment effect and 95% confidence interval.

* Heterogeneity p values

** Defined as mild, moderate or severe by trialists or by the Gross Motor Classification System (score 1 to 5).

*** Defined as developmental delay or intellectual impairment (developmental quotient or intelligence quotient more than one standard deviation below the mean), cerebral palsy (abnormality of tone with motor dysfunction), blindness or deafness, at follow up later in childhood.

† Defined as moderate or severe neurosensory impairment
