# Supplementary material for: Effects of repeat prenatal corticosteroids given to women at risk of preterm birth: An individual participant data meta-analysis
Source: PLoS Med. 2019 Apr 12;16(4):e1002771. doi: 10.1371/journal.pmed.1002771 (PMC6461224; doi:10.1371/journal.pmed.1002771)
Supplement: S3 Table — (DOCX) [file pmed.1002771.s003.docx]

**S4 Table. Secondary outcomes for the women**

| Outcome | N Trials | Repeat Corticosteroid | No Repeat Corticosteroid | RR | 95%CI | P value* |
| --- | --- | --- | --- | --- | --- | --- |
| Chorioamnionitis during labour | 10 | 134/2390 (5.6%) | 118/2346 (5.0%) | 1.12 | 0.89, 1.42 | 0.33 |
| Pyrexia after trial entry requiring the use of antibiotics | 7 | 258/1257 (20.5%) | 263/1234 (21.3%) | 0.98 | 0.84, 1.13 | 0.07 |
| Puerperal sepsis | 8 | 117/2220 (5.3%) | 105/2184 (4.8%) | 1.09 | 0.86, 1.39 | 0.11 |
| Intrapartum fever requiring the use of antibiotics | 5 | 80/1730 (4.6%) | 68/1706 (4.0%) | 1.18 | 0.86, 1.61 | 0.15 |
| Postnatal pyrexia | 4 | 79/1805 (4.4%) | 73/1780 (4.1%) | 1.07 | 0.79, 1.46 | 0.65 |
| Preterm prelabour rupture of the membranes after trial entry | 6 | 74/561 (13.2%) | 73/542 (13.5%) | 0.98 | 0.72, 1.32 | 0.86 |
| Hypertension | 4 | 167/1673 (10.0%) | 154/1653 (9.3%) | 1.07 | 0.87, 1.32 | 0.31 |
| Caesarean birth | 11 | 1381/2450 (56.4%) | 1312/2407 (54.5%) | 1.04 | 0.99, 1.09 | 0.27 |
| Postpartum haemorrhage | 3 | 152/739 (20.6%) | 149/732 (20.4%) | 1.04 | 0.86, 1.25 | 0.13 |
| Breastfeeding at hospital discharge | 1 | 350/489 (71.6%) | 363/493 (73.6%) | 0.97 | 0.90, 1.05 | N/A |
| Postnatal depression | 2 | 203/1348 (15.1%) | 209/1328 (15.7%) | 0.96 | 0.80, 1.14 | 0.23 |
| Adverse effects of repeat corticosteroid therapy** | 4 | 765/1608 (47.6%) | 787/1582 (49.7%) | 0.99 | 0.94, 1.04 | <.0001 |

Figures are numbers (percentages) with relative risk (RR) and 95% confidence interval (CI)

N/A not applicable.

* Heterogeneity p values

** Adverse effects of repeat corticosteroid therapy including gastrointestinal upset, glucose intolerance, insomnia, pain at the injection site, bruising at the injection site, infection at injection site, weight gain, Cushing appearance.
