# Supplementary material for: Effects of repeat prenatal corticosteroids given to women at risk of preterm birth: An individual participant data meta-analysis
Source: PLoS Med. 2019 Apr 12;16(4):e1002771. doi: 10.1371/journal.pmed.1002771 (PMC6461224; doi:10.1371/journal.pmed.1002771)
Supplement: S4 Table — PTB, preterm birth. (DOCX) [file pmed.1002771.s004.docx]

**S5 Table. Subgroup analysis of treatment effects by reason woman was considered to be at risk of preterm birth**

| **Outcome** | **Subgroup reason for risk of preterm birth** | **Treatment effect in those with subgroup reason for preterm birth** | **Treatment effect in those without subgroup reason for preterm birth** | **P value*** |
| --- | --- | --- | --- | --- |
| Serious outcome for infant** | Preterm labour | 1.07 (0.88, 1.29) | 0.83 (0.72, 0.97) | 0.0503 |
|  | Placenta abruption | 1.13 (0.79, 1.61) | 0.84 (0.72, 0.97) | 0.12 |
|  | Placenta previa | 0.52 (0.28, 0.95) | 0.84 (0.71, 0.99) | 0.14 |
|  | Ruptured membranes | 0.91 (0.77, 1.08) | 0.94 (0.81, 1.09) | 1.00 |
|  | Antepartum haemorrhage | 0.82 (0.59, 1.12) | 0.89 (0.77, 1.02) | 0.50 |
|  | Pre-eclampsia and/or eclampsia | 1.09 (0.81, 1.48) | 0.9 (0.79, 1.02) | 0.25 |
|  | Fetal growth restriction | 0.87 (0.64, 1.18) | 0.93 (0.81, 1.05) | 0.83 |
|  | Suspected fetal jeopardy | 1.12 (0.88, 1.42) | 0.89 (0.75, 1.05) | 0.12 |
|  | Cervical incompetence | 0.89 (0.64, 1.23) | 0.87 (0.74, 1.02) | 0.89 |
|  | Maternal disease | 1.04 (0.73, 1.48) | 0.88 (0.71, 1.1) | 0.42 |
|  | Multiple pregnancy | 1.04 (0.82, 1.32) | 0.89 (0.78, 1.01) | 0.19 |
| Use of respiratory support*** | Preterm labour | 1.01 (0.89, 1.15) | 0.86 (0.79, 0.94) | 0.07 |
|  | Placenta abruption | 0.91 (0.73, 1.12) | 0.93 (0.85, 1.01) | 0.95 |
|  | Placenta previa | 0.78 (0.57, 1.07) | 0.91 (0.82, 1.01) | 0.45 |
|  | Ruptured membranes | 0.98 (0.89, 1.08) | 0.89 (0.81, 0.97) | 0.07 |
|  | Antepartum haemorrhage | 0.89 (0.73, 1.09) | 0.91 (0.83, 1.00) | 0.65 |
|  | Pre-eclampsia and/or eclampsia | 0.84 (0.71, 0.99) | 0.93 (0.86, 1.01) | 0.63 |
|  | Fetal growth restriction | 0.81 (0.70, 0.95) | 0.93 (0.86, 1.00) | 0.49 |
|  | Suspected fetal jeopardy | 0.93 (0.82, 1.06) | 0.9 (0.81, 1.01) | 0.57 |
|  | Cervical incompetence | 0.85 (0.67, 1.08) | 0.9 (0.82, 1.00) | 0.57 |
|  | Maternal disease | 0.89 (0.68, 1.18) | 0.86 (0.74, 1.00) | 0.88 |
|  | Multiple pregnancy | 0.87 (0.75, 1.00) | 0.91 (0.85, 0.99) | 0.77 |
| Death or any neurosens-ory disability | Preterm labour | 1.09 (0.96, 1.25) | 0.98 (0.86, 1.12) | 0.25 |
|  | Placenta abruption | 1.83 (0.87, 3.88) | 0.99 (0.88, 1.13) | 0.12 |
|  | Placenta previa | 0.73 (0.48, 1.11) | 1.05 (0.92, 1.19) | 0.10 |
|  | Ruptured membranes | 1.12 (0.95, 1.31) | 1.01 (0.90, 1.12) | 0.23 |
|  | Antepartum haemorrhage | 1.03 (0.81, 1.30) | 1.04 (0.94, 1.15) | 0.95 |
|  | Pre-eclampsia and/or eclampsia | 1.10 (0.79, 1.53) | 1.03 (0.94, 1.14) | 0.77 |
|  | Fetal growth restriction | 1.13 (0.85, 1.51) | 1.03 (0.93, 1.13) | 0.62 |
|  | Suspected fetal jeopardy | 1.24 (0.92, 1.67) | 1.03 (0.91, 1.18) | 0.28 |
|  | Cervical incompetence | 1.03 (0.85, 1.24) | 1.03 (0.93, 1.15) | 0.98 |
|  | Maternal disease | 1.16 (0.87, 1.55) | 1.03 (0.90, 1.18) | 0.43 |
|  | Multiple pregnancy | 0.99 (0.80, 1.22) | 1.05 (0.94, 1.16) | 0.67 |
| Any neurosens-ory disability | Preterm labour | 1.08 (0.94, 1.24) | 0.98 (0.85, 1.13) | 0.33 |
|  | Placenta abruption | 1.54 (0.68, 3.47) | 0.98 (0.86, 1.12) | 0.27 |
|  | Placenta previa | 0.78 (0.51, 1.20) | 1.03 (0.89, 1.18) | 0.22 |
|  | Ruptured membranes | 1.13 (0.93, 1.38) | 1.00 (0.89, 1.13) | 0.23 |
|  | Antepartum haemorrhage | 1.04 (0.81, 1.33) | 1.03 (0.92, 1.15) | 1.00 |
|  | Pre-eclampsia and/or eclampsia | 0.95 (0.65, 1.39) | 1.04 (0.94, 1.15) | 0.66 |
|  | Fetal growth restriction | 1.20 (0.83, 1.74) | 1.01 (0.91, 1.13) | 0.44 |
|  | Suspected fetal jeopardy | 1.44 (0.96, 2.16) | 1.02 (0.89, 1.16) | 0.11 |
|  | Cervical incompetence | 0.99 (0.81, 1.21) | 1.04 (0.93, 1.17) | 0.72 |
|  | Maternal disease | 1.19 (0.86, 1.65) | 1.03 (0.89, 1.18) | 0.38 |
|  | Multiple pregnancy | 0.94 (0.75, 1.18) | 1.05 (0.94, 1.18) | 0.41 |
| Develop-mental delay/ intellectual impairment | Preterm labour | 1.05 (0.91, 1.22) | 0.99 (0.84, 1.16) | 0.54 |
|  | Placenta abruption | 1.40 (0.54, 3.63) | 0.98 (0.85, 1.14) | 0.45 |
|  | Placenta previa | 0.86 (0.53, 1.40) | 1.01 (0.87, 1.17) | 0.54 |
|  | Ruptured membranes | 1.13 (0.90, 1.42) | 1.00 (0.88, 1.13) | 0.31 |
|  | Antepartum haemorrhage | 1.05 (0.80, 1.39) | 1.02 (0.91, 1.14) | 0.87 |
|  | Pre-eclampsia and/or eclampsia | 0.98 (0.64, 1.50) | 1.02 (0.92, 1.15) | 0.84 |
|  | Fetal growth restriction | 1.29 (0.86, 1.92) | 1.00 (0.89, 1.12) | 0.24 |
|  | Suspected fetal jeopardy | 1.54 (0.99, 2.37) | 0.99 (0.86, 1.14) | 0.06 |
|  | Cervical incompetence | 0.94 (0.76, 1.15) | 1.05 (0.93, 1.20) | 0.37 |
|  | Maternal disease | 1.44 (1.01, 2.05) | 0.97 (0.84, 1.12) | <0.05 |
|  | Multiple pregnancy | 0.90 (0.71, 1.14) | 1.05 (0.93, 1.19) | 0.26 |
| Chronic lung disease | Preterm labour | 1.26 (0.77, 2.06) | 0.89 (0.69, 1.13) | 0.21 |
|  | Placenta abruption | 1.19 (0.56, 2.56) | 0.92 (0.73, 1.16) | 0.49 |
|  | Placenta previa | 0.79 (0.33, 1.86) | 0.94 (0.74, 1.19) | 0.74 |
|  | Ruptured membranes | 0.94 (0.70, 1.27) | 1.08 (0.84, 1.40) | 0.55 |
|  | Antepartum haemorrhage | 1.23 (0.74, 2.04) | 0.91 (0.72, 1.16) | 0.61 |
|  | Pre-eclampsia and/or eclampsia | 0.83 (0.46, 1.50) | 0.98 (0.78, 1.22) | 0.63 |
|  | Fetal growth restriction | 1.04 (0.55, 1.99) | 0.95 (0.76, 1.18) | 0.83 |
|  | Suspected fetal jeopardy | 1.90 (0.83, 4.34) | 0.90 (0.66, 1.24) | 0.08 |
|  | Cervical incompetence | 0.72 (0.38, 1.36) | 0.97 (0.75, 1.26) | 0.34 |
|  | Maternal disease | 1.09 (0.50, 2.35) | 0.95 (0.61, 1.48) | 0.68 |
|  | Multiple pregnancy | 1.09 (0.70, 1.68) | 0.97 (0.78, 1.21) | 0.61 |
| Death at any time | Preterm labour | 1.28 (0.86, 1.9) | 0.80 (0.55, 1.15) | 0.08 |
|  | Placenta abruption | 2.41 (0.26, 21.9) | 0.95 (0.65, 1.37) | 0.43 |
|  | Ruptured membranes | 1.01 (0.68, 1.51) | 0.90 (0.64, 1.27) | 0.67 |
|  | Antepartum haemorrhage | 0.88 (0.40, 1.92) | 0.93 (0.69, 1.25) | 0.86 |
|  | Pre-eclampsia and/or eclampsia | 1.01 (0.44, 2.34) | 0.97 (0.73, 1.29) | 0.99 |
|  | Fetal growth restriction | 0.71 (0.39, 1.29) | 1.01 (0.75, 1.36) | 0.43 |
|  | Suspected fetal jeopardy | 0.99 (0.56, 1.75) | 0.98 (0.67, 1.44) | 0.99 |
|  | Cervical incompetence | 1.48 (0.71, 3.09) | 0.88 (0.64, 1.21) | 0.21 |
|  | Maternal disease | 1.04 (0.51, 2.14) | 0.94 (0.63, 1.39) | 0.82 |
|  | Multiple pregnancy | 1.38 (0.79, 2.41) | 0.88 (0.65, 1.18) | 0.15 |
| Maternal sepsis | Preterm labour | 1.11 (0.90, 1.37) | 0.92 (0.80, 1.07) | 0.16 |
|  | Placenta abruption | 1.07 (0.62, 1.84) | 0.98 (0.86, 1.12) | 0.61 |
|  | Placenta previa | 0.73 (0.49, 1.08) | 1.01 (0.88, 1.17) | 0.14 |
|  | Ruptured membranes | 1.13 (0.97, 1.32) | 0.93 (0.79, 1.10) | 0.12 |
|  | Antepartum haemorrhage | 0.93 (0.71, 1.20) | 1.00 (0.86, 1.16) | 0.59 |
|  | Pre-eclampsia and/or eclampsia | 0.71 (0.45, 1.12) | 1.03 (0.91, 1.17) | 0.15 |
|  | Fetal growth restriction | 0.64 (0.35, 1.18) | 1.03 (0.91, 1.17) | 0.11 |
|  | Suspected fetal jeopardy | 0.99 (0.61, 1.61) | 1.11 (0.91, 1.36) | 0.63 |
|  | Cervical incompetence | 1.04 (0.70, 1.55) | 0.94 (0.80, 1.09) | 0.73 |
|  | Maternal disease | 1.37 (0.82, 2.31) | 0.92 (0.68, 1.23) | 0.18 |
|  | Multiple pregnancy | 0.92 (0.63, 1.35) | 1.00 (0.88, 1.14) | 0.80 |
| Birth weight (g)# | Preterm labour | -134 (-195, -73) | -23 (-93, 47) | 0.03 |
|  | Placenta abruption | 18 (-181, 217) | -69 (-134, -4) | 0.48 |
|  | Placenta previa | 46 (-143, 234) | -83 (-155, -12) | 0.16 |
|  | Ruptured membranes | -100 (-178, -22) | -78 (-128, -28) | 0.62 |
|  | Antepartum haemorrhage | -25 (-161, 111) | -109 (-162, -56) | 0.14 |
|  | Pre-eclampsia and/or eclampsia | -11 (-150, 127) | -97 (-146, -47) | 0.30 |
|  | Fetal growth restriction | 48 (-68, 163) | -91 (-140, -43) | 0.04 |
|  | Suspected fetal jeopardy | -46 (-167, 75) | -118 (-175, -61) | 0.36 |
|  | Cervical incompetence | -122 (-215, -28) | -71 (-134, -8) | 0.41 |
|  | Maternal disease | -76 (-21, 65) | -128 (-194, -62) | 0.53 |
|  | Multiple pregnancy | -100 (-171, -30) | -76 (-127, -25) | 0.41 |
| Head circumfer-ence at birth (cm)# | Preterm labour | -0.63 (-0.9, -0.4) | -0.01 (-0.3, 0.3) | 0.004 |
|  | Placenta abruption | -0.19 (-1.2, 0.8) | -0.19 (-0.5, 0.1) | 0.98 |
|  | Placenta previa | -0.21 (-1.0, 0.6) | -0.22 (-0.5, 0.1) | 0.77 |
|  | Ruptured membranes | -0.52 (-1.0, -0.1) | -0.32 (-0.5, -0.1) | 0.44 |
|  | Antepartum haemorrhage | -0.32 (-0.9, 0.3) | -0.41 (-0.6, -0.2) | 0.57 |
|  | Pre-eclampsia and/or eclampsia | -0.02 (-0.7, 0.6) | -0.39 (-0.6, -0.2) | 0.37 |
|  | Fetal growth restriction | -0.02 (-0.7, 0.6) | -0.37 (-0.6, -0.2) | 0.33 |
|  | Suspected fetal jeopardy | -0.35 (-0.9, 0.2) | -0.46 (-0.7, -0.2) | 0.79 |
|  | Cervical incompetence | -0.53 (-0.9, -0.2) | -0.32 (-0.6, -0.1) | 0.37 |
|  | Maternal disease | -0.42 (-1.0, 0.2) | -0.52 (-0.8, -0.3) | 0.72 |
|  | Multiple pregnancy | -0.58 (-0.9, -0.2) | -0.27 (-0.5, -0.1) | 0.08 |
| Length at birth (cm)# | Preterm labour | -0.95 (-1.4, -0.5) | -0.07 (-0.6, 0.4) | 0.01 |
|  | Placenta abruption | -0.14 (-1.7, 1.4) | -0.44 (-0.9, 0.01) | 0.68 |
|  | Placenta previa | 0.13 (-1.2, 1.4) | -0.51 (-1.0, -0.02) | 0.31 |
|  | Ruptured membranes | -0.69 (-1.3, -0.04) | -0.63 (-1.0, -0.3) | 0.82 |
|  | Antepartum haemorrhage | -0.35 (-1.3, 0.6) | -0.69 (-1.1, -0.3) | 0.33 |
|  | Pre-eclampsia and/or eclampsia | 0.32 (-0.7, 1.4) | -0.69 (-1.0, -0.4) | 0.09 |
|  | Fetal growth restriction | 0.39 (-0.6, 1.4) | -0.64 (-1.0, -0.3) | 0.08 |
|  | Suspected fetal jeopardy | -0.31 (-1.2, 0.6) | -0.81 (-1.2, -0.4) | 0.41 |
|  | Cervical incompetence | -1.07 (-1.7, -0.5) | -0.34 (-0.8, 0.1) | 0.06 |
|  | Maternal disease | -0.15 (-1.1, 0.7) | -0.94 (-1.4, -0.5) | 0.12 |
|  | Multiple pregnancy | -0.98 (-1.6, -0.4) | -0.53 (-0.9, -0.2) | 0.15 |

Figures are relative risk or # adjusted mean difference and 95% confidence interval.

*P values from interaction term: compares treatment effect in the subgroup with and without the specified reason for preterm birth.

** defined by the Precise Group as any death [fetal, neonatal, infant or child], severe respiratory disease as defined by the trialists, grade 3 or 4 intraventricular haemorrhage [IVH], chronic lung disease [oxygen dependent at 36 weeks’ postmenstrual age], definite necrotising enterocolitis, stage 3 or worse retinopathy of prematurity in the better eye, or cystic periventricular leukomalacia.

*** defined as use of mechanical ventilation or continuous positive airways pressure or other respiratory support.
