# Supplementary material for: Effects of repeat prenatal corticosteroids given to women at risk of preterm birth: An individual participant data meta-analysis
Source: PLoS Med. 2019 Apr 12;16(4):e1002771. doi: 10.1371/journal.pmed.1002771 (PMC6461224; doi:10.1371/journal.pmed.1002771)
Supplement: S5 Table — (DOCX) [file pmed.1002771.s005.docx]

**S6 Table. Subgroup analysis of treatment effects among the subgroups – singleton versus multiple birth**

| **Outcome** | **Number of fetuses in utero** | **Treatment effect** | **LCL** | **UCL** | **P value*** |
| --- | --- | --- | --- | --- | --- |
| Serious outcome for infant** | 1 | 0.92 | 0.80 | 1.05 | 0.30 |
|  | 2 | 1.02 | 0.81 | 1.29 |  |
|  | ≥3 | 0.67 | 0.38 | 1.20 |  |
| Use of respiratory support*** | 1 | 0.92 | 0.85 | 1.00 | 0.61 |
|  | 2 | 0.87 | 0.75 | 1.00 |  |
|  | ≥3 | 0.84 | 0.59 | 1.18 |  |
| Death or any neurosensory disability | 1 | 1.04 | 0.94 | 1.16 | 0.82 |
|  | 2 | 1.03 | 0.85 | 1.25 |  |
|  | ≥3 | 0.90 | 0.59 | 1.37 |  |
| Any neurosensory disability | 1 | 1.05 | 0.93 | 1.18 | 0.82 |
|  | 2 | 1.00 | 0.81 | 1.23 |  |
|  | ≥3 | 0.94 | 0.61 | 1.44 |  |
| Developmental delay/ intellectual impairment | 1 | 1.06 | 0.93 | 1.20 | 0.53 |
|  | 2 | 0.92 | 0.73 | 1.16 |  |
|  | ≥3 | 0.99 | 0.63 | 1.56 |  |
| Chronic lung disease | 1 | 0.99 | 0.78 | 1.25 | 0.84 |
|  | ≥2 | 1.03 | 0.71 | 1.50 |  |
| Death at any time | 1 | 0.87 | 0.64 | 1.19 | 0.39 |
|  | 2 | 1.29 | 0.79 | 2.12 |  |
|  | ≥3 | 1.41 | 0.36 | 5.43 |  |
| Maternal sepsis | 1 | 1.01 | 0.89 | 1.15 | 0.34 |
|  | 2 | 0.93 | 0.67 | 1.30 |  |
|  | ≥3 | 0.42 | 0.12 | 1.54 |  |
| Birthweight (g)# | 1 | -88 | -140 | -36 | 0.38 |
|  | 2 | -82 | -156 | -9 |  |
|  | ≥3 | 17 | -122 | 157 |  |
| Head circumference at birth (cm)# | 1 | -0.31 | -0.53 | -0.09 | 0.47 |
|  | 2 | -0.51 | -0.87 | -0.14 |  |
|  | ≥3 | -0.07 | -0.91 | 0.77 |  |
| Length at birth (cm)# | 1 | -0.60 | -0.97 | -0.23 | 0.63 |
|  | 2 | -0.82 | -1.48 | -0.17 |  |
|  | ≥3 | -0.30 | -1.71 | 1.12 |  |

Figures are relative risk (RR) or # adjusted mean difference as treatment effect and 95% confidence interval. LCL = 95% Lower confidence limit; UCL = 95% Upper confidence limit.

*P values for heterogeneity.

** defined by the Precise Group as any death [fetal, neonatal, infant or child], severe respiratory disease as defined by the trialists, grade 3 or 4 intraventricular haemorrhage [IVH], chronic lung disease [oxygen dependent at 36 weeks’ postmenstrual age], definite necrotising enterocolitis, stage 3 or worse retinopathy of prematurity in the better eye, or cystic periventricular leukomalacia.

*** defined as use of mechanical ventilation or continuous positive airways pressure or other respiratory support.
