# Supplementary material for: Effects of repeat prenatal corticosteroids given to women at risk of preterm birth: An individual participant data meta-analysis
Source: PLoS Med. 2019 Apr 12;16(4):e1002771. doi: 10.1371/journal.pmed.1002771 (PMC6461224; doi:10.1371/journal.pmed.1002771)
Supplement: S6 Table — GA, gestational age. (DOCX) [file pmed.1002771.s006.docx]

**S7 Table. Subgroup analysis of treatment effects among the subgroups of gestational age (weeks) when first repeat treatment course was given**

| **Outcome** | **Gestational age when first trial treatment course was given (weeks)** | **Treatment effect** | **LCL** | **UCL** | **P (linear trend)** |
| --- | --- | --- | --- | --- | --- |
| Serious outcome for infant** | <26 | 0.81 | 0.66 | 1.00 | 0.98 |
|  | 26 to <28 | 1.01 | 0.86 | 1.19 |  |
|  | 28 to <30 | 0.93 | 0.74 | 1.17 |  |
|  | 30 to <32 | 0.83 | 0.60 | 1.15 |  |
|  | 32 to <34 | 0.70 | 0.37 | 1.30 |  |
| Use of respiratory support*** | <26 | 0.90 | 0.77 | 1.04 | 0.98 |
|  | 26 to <28 | 1.01 | 0.89 | 1.14 |  |
|  | 28 to <30 | 1.03 | 0.93 | 1.15 |  |
|  | 30 to <32 | 0.79 | 0.68 | 0.93 |  |
|  | 32 to <34 | 0.71 | 0.53 | 0.96 |  |
| Death or any neurosensory disability | <26 | 1.01 | 0.80 | 1.28 | 0.74 |
|  | 26 to <28 | 1.06 | 0.90 | 1.25 |  |
|  | 28 to <30 | 1.05 | 0.88 | 1.25 |  |
|  | 30 to <32 | 0.99 | 0.82 | 1.20 |  |
|  | 32 to <34 | 0.97 | 0.62 | 1.51 |  |
| Any neurosensory disability | <26 | 1.02 | 0.77 | 1.35 | 0.62 |
|  | 26 to <28 | 1.09 | 0.91 | 1.31 |  |
|  | 28 to <30 | 1.01 | 0.84 | 1.22 |  |
|  | 30 to <32 | 0.99 | 0.81 | 1.21 |  |
|  | 32 to <34 | . | . | . |  |
| Developmental delay/  intellectual impairment | <26 | 1.05 | 0.78 | 1.43 | 0.46 |
|  | 26 to <28 | 1.12 | 0.91 | 1.37 |  |
|  | 28 to <30 | 0.93 | 0.76 | 1.14 |  |
|  | 30 to <32 | 0.99 | 0.80 | 1.23 |  |
|  | 32 to <34 | . | . | . |  |
| Chronic lung disease | <26 | 1.01 | 0.76 | 1.36 | 0.46 |
|  | 26 to <28 | 1.18 | 0.88 | 1.59 |  |
|  | 28 to <30 | 0.87 | 0.53 | 1.41 |  |
|  | 30 to <32 | 0.69 | 0.29 | 1.64 |  |
|  | 32 to <34 | 0.55 | 0.04 | 7.73 |  |
| Death at any time | <26 | 0.96 | 0.57 | 1.60 | 0.91 |
|  | 26 to <28 | 0.93 | 0.61 | 1.43 |  |
|  | 28 to <30 | 1.17 | 0.69 | 1.98 |  |
|  | 30 to <32 | 1.05 | 0.52 | 2.15 |  |
|  | 32 to <34 | 0.69 | 0.18 | 2.60 |  |
| Maternal sepsis | <26 | 1.04 | 0.82 | 1.33 | 0.62 |
|  | 26 to <28 | 1.07 | 0.85 | 1.35 |  |
|  | 28 to <30 | 0.94 | 0.73 | 1.21 |  |
|  | 30 to <32 | 0.83 | 0.64 | 1.08 |  |
|  | 32 to <34 | 1.74 | 0.96 | 3.15 |  |
| Birthweight (z-scores)# | <26 | -0.31 | -0.49 | -0.12 | 0.004 |
|  | 26 to <28 | -0.21 | -0.32 | -0.09 |  |
|  | 28 to <30 | -0.13 | -0.24 | -0.02 |  |
|  | 30 to <32 | -0.03 | -0.14 | 0.07 |  |
|  | 32 to <34 | -0.02 | -0.19 | 0.16 |  |
| Head circumference at birth (z-scores)# | <26 | -0.27 | -0.47 | -0.07 | 0.026 |
|  | 26 to <28 | -0.29 | -0.42 | -0.16 |  |
|  | 28 to <30 | -0.09 | -0.21 | 0.03 |  |
|  | 30 to <32 | -0.07 | -0.19 | 0.05 |  |
|  | 32 to <34 | -0.11 | -0.29 | 0.08 |  |
| Length at birth (z-scores)# | <26 | -0.27 | -0.47 | -0.06 | 0.05 |
|  | 26 to <28 | -0.24 | -0.38 | -0.09 |  |
|  | 28 to <30 | -0.08 | -0.22 | 0.06 |  |
|  | 30 to <32 | -0.07 | -0.21 | 0.07 |  |
|  | 32 to <34 | -0.08 | -0.30 | 0.13 |  |

Figures are relative risk (RR) or # adjusted mean difference as treatment effect and 95% confidence interval. LCL = 95% Lower confidence limit; UCL = 95% Upper confidence limit.

P values for linear trend.

** defined by the Precise Group as any death [fetal, neonatal, infant or child], severe respiratory disease as defined by the trialists, grade 3 or 4 intraventricular haemorrhage [IVH], chronic lung disease [oxygen dependent at 36 weeks’ postmenstrual age], definite necrotising enterocolitis, stage 3 or worse retinopathy of prematurity in the better eye, or cystic periventricular leukomalacia.

*** defined as use of mechanical ventilation or continuous positive airways pressure or other respiratory support.
