# Supplementary material for: Effects of repeat prenatal corticosteroids given to women at risk of preterm birth: An individual participant data meta-analysis
Source: PLoS Med. 2019 Apr 12;16(4):e1002771. doi: 10.1371/journal.pmed.1002771 (PMC6461224; doi:10.1371/journal.pmed.1002771)
Supplement: S7 Table — (DOCX) [file pmed.1002771.s007.docx]

**S8 Table. Subgroup analysis of treatment effects among the subgroups according to time (days) prior to birth last dose of trial treatment course was given**

| **Outcome** | **Time prior to birth last dose of trial treatment course was given (days)** | **Treatment effect** | **LCL** | **UCL** | **P value*** |
| --- | --- | --- | --- | --- | --- |
| Serious outcome for infant** | <7 | 0.86 | 0.74 | 1.00 | 0.67 |
|  | 7 to <14 | 0.95 | 0.70 | 1.30 |  |
|  | ≥14 | 0.73 | 0.44 | 1.23 |  |
| Use of respiratory support*** | <7 | 0.90 | 0.84 | 0.97 | 0.35 |
|  | 7 to <14 | 0.81 | 0.65 | 1.00 |  |
|  | ≥14 | 0.78 | 0.59 | 1.02 |  |
| Death or any neurosensory disability | <7 | 0.99 | 0.86 | 1.13 | 0.79 |
|  | 7 to <14 | 1.01 | 0.80 | 1.28 |  |
|  | ≥14 | 1.06 | 0.91 | 1.23 |  |
| Any neurosensory disability | <7 | 1.02 | 0.88 | 1.19 | 0.91 |
|  | 7 to <14 | 0.98 | 0.75 | 1.28 |  |
|  | ≥14 | 1.04 | 0.89 | 1.21 |  |
| Developmental delay/ intellectual impairment | <7 | 1.04 | 0.87 | 1.23 | 0.97 |
|  | 7 to <14 | 0.99 | 0.73 | 1.34 |  |
|  | ≥14 | 1.00 | 0.86 | 1.18 |  |
| Chronic lung disease | <7 | 0.99 | 0.79 | 1.26 | 0.77 |
|  | 7 to <14 | 0.79 | 0.40 | 1.53 |  |
|  | ≥14 | 1.16 | 0.46 | 2.96 |  |
| Death at any time | <7 | 0.79 | 0.55 | 1.13 | 0.23 |
|  | 7 to <14 | 1.15 | 0.62 | 2.13 |  |
|  | ≥14 | 1.62 | 0.70 | 3.72 |  |
| Maternal sepsis | <7 | 0.85 | 0.71 | 1.01 | 0.22 |
|  | 7 to <14 | 1.13 | 0.76 | 1.68 |  |
|  | ≥14 | 1.06 | 0.81 | 1.39 |  |
| Birthweight (z-scores)# | <7 | -0.12 | -0.22 | -0.01 | 0.86 |
|  | 7 to <14 | -0.08 | -0.25 | 0.09 |  |
|  | ≥14 | -0.13 | -0.21 | -0.05 |  |
| Head circumference at birth (z-scores)# | <7 | -0.16 | -0.28 | -0.05 | 0.41 |
|  | 7 to <14 | -0.27 | -0.44 | -0.09 |  |
|  | ≥14 | -0.13 | -0.23 | -0.04 |  |
| Length at birth (z-scores)# | <7 | -0.11 | -0.24 | 0.01 | 0.96 |
|  | 7 to <14 | -0.12 | -0.33 | 0.09 |  |
|  | ≥14 | -0.13 | -0.24 | -0.02 |  |

Figures are relative risk (RR) or # adjusted mean difference as treatment effect and 95% confidence interval. LCL = 95% Lower confidence limit; UCL = 95% Upper confidence limit.

*P values for subgroup comparisons.

** defined by the Precise Group as any death [fetal, neonatal, infant or child], severe respiratory disease as defined by the trialists, grade 3 or 4 intraventricular haemorrhage [IVH], chronic lung disease [oxygen dependent at 36 weeks’ postmenstrual age], definite necrotising enterocolitis, stage 3 or worse retinopathy of prematurity in the better eye, or cystic periventricular leukomalacia.

*** defined as use of mechanical ventilation or continuous positive airways pressure or other respiratory support.
