# Supplementary material for: Effects of repeat prenatal corticosteroids given to women at risk of preterm birth: An individual participant data meta-analysis
Source: PLoS Med. 2019 Apr 12;16(4):e1002771. doi: 10.1371/journal.pmed.1002771 (PMC6461224; doi:10.1371/journal.pmed.1002771)
Supplement: S8 Table — (DOCX) [file pmed.1002771.s008.docx]

**S9 Table. Subgroup analysis of treatment effects among the subgroups considered by number of repeat courses of treatment received**

| **Outcome** | **Number of repeat courses of treatment received** | **Treatment effect** | **LCL** | **UCL** | **P*** |
| --- | --- | --- | --- | --- | --- |
| Serious outcome** | 1 | 1.08 | 0.94 | 1.23 | <0.001 |
|  | 2-3 | 0.72 | 0.56 | 0.91 |  |
|  | 4-5 | 0.46 | 0.29 | 0.74 |  |
|  | ≥6 | 1.64 | 0.67 | 4.03 |  |
| Use of respiratory support*** | 1 | 0.98 | 0.91 | 1.06 | 0.005 |
|  | 2-3 | 0.81 | 0.70 | 0.94 |  |
|  | 4-5 | 0.61 | 0.45 | 0.82 |  |
|  | ≥6 | 0.97 | 0.57 | 1.66 |  |
| Death or any neuro-sensory disability | 1 | 1.08 | 0.94 | 1.24 | 0.22 |
|  | 2-3 | 0.93 | 0.79 | 1.08 |  |
|  | 4-5 | 1.02 | 0.82 | 1.27 |  |
|  | ≥6 | 1.49 | 0.84 | 2.63 |  |
| Any neuro-sensory disability | 1 | 1.03 | 0.88 | 1.21 | 0.62 |
|  | 2-3 | 0.98 | 0.83 | 1.16 |  |
|  | 4-5 | 1.02 | 0.82 | 1.27 |  |
|  | ≥6 | 1.45 | 0.82 | 2.54 |  |
| Developmental delay/  intellectual impairment | 1 | 1.05 | 0.88 | 1.25 | 0.43 |
|  | 2-3 | 0.94 | 0.78 | 1.13 |  |
|  | 4-5 | 0.99 | 0.79 | 1.25 |  |
|  | ≥6 | 1.66 | 0.86 | 3.21 |  |
| Chronic lung disease | 1 | 1.01 | 0.79 | 1.28 | 0.61 |
|  | 2-3 | 1.08 | 0.74 | 1.58 |  |
|  | 4-5 | 0.56 | 0.27 | 1.18 |  |
|  | ≥6 | 1.73 | 0.45 | 6.67 |  |
| Maternal sepsis | 1 | 0.88 | 0.75 | 1.05 | 0.2788 |
|  | 2-3 | 1.17 | 0.92 | 1.48 | . |
|  | 4-5 | 0.98 | 0.70 | 1.37 | . |
|  | ≥6 | 1.18 | 0.73 | 1.89 | . |
| Birthweight (z-scores)# | 1 | -0.09 | -0.18 | -0.01 | 0.001 |
|  | 2-3 | -0.03 | -0.13 | 0.08 |  |
|  | 4-5 | -0.26 | -0.40 | -0.11 |  |
|  | ≥6 | -0.57 | -0.83 | -0.32 |  |
| Head circumference at birth (z-scores)# | 1 | -0.07 | -0.16 | 0.03 | 0.008 |
|  | 2-3 | -0.16 | -0.27 | -0.04 |  |
|  | 4-5 | -0.31 | -0.47 | -0.14 |  |
|  | ≥6 | -0.60 | -0.95 | -0.25 |  |
| Length at birth (Z-scores)# | 1 | -0.09 | -0.20 | 0.02 | 0.003 |
|  | 2-3 | -0.03 | -0.16 | 0.10 |  |
|  | 4-5 | -0.28 | -0.46 | -0.11 |  |
|  | ≥6 | -0.56 | -0.87 | -0.24 |  |

Figures are relative risk (RR) or # adjusted mean difference as treatment effect and 95% confidence interval. LCL = 95% Lower confidence limit; UCL = 95% Upper confidence limit.

*P values for subgroup comparison. P values for a linear trend in subgroup estimates were similar.

** defined by the Precise Group as any death [fetal, neonatal, infant or child], severe respiratory disease as defined by the trialists, grade 3 or 4 intraventricular haemorrhage [IVH], chronic lung disease [oxygen dependent at 36 weeks’ postmenstrual age], definite necrotising enterocolitis, stage 3 or worse retinopathy of prematurity in the better eye, or cystic periventricular leukomalacia.

*** defined as use of mechanical ventilation or continuous positive airways pressure or other respiratory support.
