# Supplementary material for: Effects of repeat prenatal corticosteroids given to women at risk of preterm birth: An individual participant data meta-analysis
Source: PLoS Med. 2019 Apr 12;16(4):e1002771. doi: 10.1371/journal.pmed.1002771 (PMC6461224; doi:10.1371/journal.pmed.1002771)
Supplement: S9 Table — (DOCX) [file pmed.1002771.s009.docx]

**S10 Table. Subgroup analysis of treatment effects among the subgroups according to minimum actual interval (days) between repeat trial treatment courses**

| **Outcome** | **Minimum interval between repeat trial treatment courses (days)** | **Treatment effect** | **LCL** | **UCL** | **P value*** |
| --- | --- | --- | --- | --- | --- |
| Serious outcome for infant** | Single course | 1.06 | 0.90 | 1.24 | 0.005 |
|  | 1-7 | 0.69 | 0.54 | 0.88 |  |
|  | ≥8 | 0.65 | 0.43 | 0.98 |  |
| Use of respiratory support*** | Single course | 1.03 | 0.92 | 1.15 | 0.002 |
|  | 1-7 | 0.78 | 0.67 | 0.91 |  |
|  | ≥8 | 0.70 | 0.52 | 0.93 |  |
| Death or any neuro-sensory disability | Single course | 1.10 | 0.95 | 1.26 | 0.52 |
|  | 1-7 | 0.97 | 0.83 | 1.14 |  |
|  | ≥8 | 1.01 | 0.84 | 1.22 |  |
| Any neurosensory disability | Single course | 1.05 | 0.90 | 1.23 | 0.85 |
|  | 1-7 | 0.99 | 0.84 | 1.17 |  |
|  | ≥8 | 1.06 | 0.87 | 1.30 |  |
| Developmental delay/ intellectual impairment | Single course | 1.07 | 0.90 | 1.27 | 0.74 |
|  | 1-7 | 0.97 | 0.81 | 1.17 |  |
|  | ≥8 | 1.03 | 0.83 | 1.27 |  |
| Death at any time | Single course | 1.28 | 0.90 | 1.84 | 0.03 |
|  | 1-7 | 0.66 | 0.35 | 1.24 |  |
|  | ≥8 | 0.52 | 0.26 | 1.03 |  |
| Maternal sepsis | Single course | 0.85 | 0.71 | 1.02 | 0.10 |
|  | 1-7 | 1.10 | 0.91 | 1.34 |  |
|  | ≥8 | 1.28 | 0.82 | 1.99 |  |
| Birthweight (Z-scores)# | Single course | -0.14 | -0.24 | -0.04 | 0.47 |
|  | 1-7 | -0.18 | -0.29 | -0.07 |  |
|  | ≥8 | -0.08 | -0.20 | 0.03 |  |
| Head circumference at birth (Z-scores)# | Single course | -0.12 | -0.24 | -0.01 | 0.32 |
|  | 1-7 | -0.21 | -0.33 | -0.09 |  |
|  | ≥8 | -0.24 | -0.37 | -0.10 |  |
| Length at birth (Z-scores)# | Single course | -0.11 | -0.24 | 0.02 | 0.51 |
|  | 1-7 | -0.19 | -0.31 | -0.06 |  |
|  | ≥8 | -0.09 | -0.24 | 0.06 |  |

Figures are relative risk (RR) or # adjusted mean difference as treatment effect and 95% confidence interval. LCL = 95% Lower confidence limit; UCL = 95% Upper confidence limit.

*P values for subgroup comparisons.

** defined by the Precise Group as any death [fetal, neonatal, infant or child], severe respiratory disease as defined by the trialists, grade 3 or 4 intraventricular haemorrhage [IVH], chronic lung disease [oxygen dependent at 36 weeks’ postmenstrual age], definite necrotising enterocolitis, stage 3 or worse retinopathy of prematurity in the better eye, or cystic periventricular leukomalacia.

*** defined as use of mechanical ventilation or continuous positive airways pressure or other respiratory support.
