# Supplementary material for: Effects of repeat prenatal corticosteroids given to women at risk of preterm birth: An individual participant data meta-analysis
Source: PLoS Med. 2019 Apr 12;16(4):e1002771. doi: 10.1371/journal.pmed.1002771 (PMC6461224; doi:10.1371/journal.pmed.1002771)
Supplement: S10 Table — (DOCX) [file pmed.1002771.s010.docx]

**S11 Table. Subgroup analysis of treatment effects among the subgroups according to dose of corticosteroids received (mg)**

| **Outcome** | **Total trial treatment received (mg)** | **Treatment effect** | **LCL** | **UCL** | **P value*** |
| --- | --- | --- | --- | --- | --- |
| Serious outcome for infant** | ≤12 | 1.06 | 0.88 | 1.29 | 0.03 |
|  | >12-24 | 1.00 | 0.84 | 1.19 |  |
|  | >24-48 | 0.70 | 0.53 | 0.93 |  |
|  | >48 | 0.67 | 0.46 | 0.97 |  |
| Use of respiratory support*** | ≤12 | 1.00 | 0.91 | 1.10 | 0.004 |
|  | >12-24 | 0.93 | 0.83 | 1.05 |  |
|  | >24-48 | 0.69 | 0.56 | 0.85 |  |
|  | >48 | 0.78 | 0.62 | 0.99 |  |
| Death or any neurosensory disability | ≤12 | 1.03 | 0.85 | 1.25 | 0.37 |
|  | >12-24 | 1.10 | 0.93 | 1.30 |  |
|  | >24-48 | 0.90 | 0.74 | 1.09 |  |
|  | >48 | 1.07 | 0.89 | 1.29 |  |
| Any neurosensory disability | ≤12 | 0.95 | 0.76 | 1.19 | 0.68 |
|  | >12-24 | 1.12 | 0.92 | 1.35 |  |
|  | >24-48 | 0.98 | 0.80 | 1.21 |  |
|  | >48 | 1.04 | 0.86 | 1.25 |  |
| Developmental delay/  intellectual impairment | ≤12 | 1.01 | 0.78 | 1.30 | 0.81 |
|  | >12-24 | 1.08 | 0.88 | 1.33 |  |
|  | >24-48 | 0.96 | 0.77 | 1.19 |  |
|  | >48 | 1.03 | 0.85 | 1.24 |  |
| Chronic lung disease | ≤12 | 1.05 | 0.74 | 1.48 | 0.87 |
|  | >12-24 | 0.92 | 0.68 | 1.26 |  |
|  | >24-48 | 1.09 | 0.71 | 1.68 |  |
|  | >48 | 0.80 | 0.42 | 1.52 |  |
| Death at any time | ≤12 | 1.85 | 0.99 | 3.46 | <0.001 |
|  | >12-24 | 0.88 | 0.60 | 1.29 |  |
|  | >24-48 | 0.33 | 0.15 | 0.72 |  |
|  | >48 | 2.11 | 0.87 | 5.11 |  |
| Maternal sepsis | ≤12 | 0.87 | 0.71 | 1.07 | 0.26 |
|  | >12-24 | 1.02 | 0.81 | 1.29 |  |
|  | >24-48 | 0.99 | 0.74 | 1.33 |  |
|  | >48 | 1.18 | 0.89 | 1.56 |  |
| Birthweight (Z-scores)# | ≤12 | -0.10 | -0.24 | 0.04 | 0.31 |
|  | >12-24 | -0.05 | -0.14 | 0.05 |  |
|  | >24-48 | -0.19 | -0.32 | -0.05 |  |
|  | >48 | -0.16 | -0.27 | -0.05 |  |
| Head circumference at birth (Z-scores)# | ≤12 | -0.07 | -0.21 | 0.08 | 0.04 |
|  | >12-24 | -0.07 | -0.18 | 0.05 |  |
|  | >24-48 | -0.26 | -0.41 | -0.11 |  |
|  | >48 | -0.26 | -0.38 | -0.13 |  |
| Length at birth (Z-scores)# | ≤12 | -0.10 | -0.25 | 0.06 | 0.26 |
|  | >12-24 | -0.02 | -0.15 | 0.12 |  |
|  | >24-48 | -0.15 | -0.31 | 0.02 |  |
|  | >48 | -0.21 | -0.34 | -0.08 |  |

Figures are relative risk (RR) or # adjusted mean difference as treatment effect and 95% confidence interval. LCL = 95% Lower confidence limit; UCL = 95% Upper confidence limit.

*P values for subgroup differences.

** defined by the Precise Group as any death [fetal, neonatal, infant or child], severe respiratory disease as defined by the trialists, grade 3 or 4 intraventricular haemorrhage [IVH], chronic lung disease [oxygen dependent at 36 weeks’ postmenstrual age], definite necrotising enterocolitis, stage 3 or worse retinopathy of prematurity in the better eye, or cystic periventricular leukomalacia.

*** defined as use of mechanical ventilation or continuous positive airways pressure or other respiratory support.
