# Supplementary material for: Effects of repeat prenatal corticosteroids given to women at risk of preterm birth: An individual participant data meta-analysis
Source: PLoS Med. 2019 Apr 12;16(4):e1002771. doi: 10.1371/journal.pmed.1002771 (PMC6461224; doi:10.1371/journal.pmed.1002771)
Supplement: S2 Text — Repeat prenatal corticosteroid prior to preterm birth: a systematic review and IPD-MA for the PRECISE study group Study Protocol. IPD, individual participant data; MA, meta-analysis; PRECISE, Prenatal Repeat Corticosteroid International IPD-MA Study: assessing the effects using the best level of Evidence. (PDF) [file pmed.1002771.s012.pdf]

Study Protocol

Repeat prenatal corticosteroid prior to preterm birth: a systematic review and individual patient data meta-analysis for the PRECISE Study Group (Prenatal Repeat Corticosteroid International IPD Study Group: assessing the effects using the best level of Evidence) for the PRECISE Collaboration.

[individuals names to be added when confirmed for each of the trials]

Version: 7/4/11

CONFIDENTIAL DRAFT

Aims

The aim of this individual patient data meta-analysis is to assess whether the effects of repeat prenatal corticosteroid treatment given to women at risk of preterm birth are modified in a clinically meaningful way by factors related to the women or trial protocol.

The Prenatal Repeat Corticosteroid International IPD Study Group: assessing the effects using the best level of Evidence (PRECISE) Collaboration will conduct an individual patient data meta-analysis.

Background

Clinical significance of respiratory distress syndrome and other neonatal morbidities in preterm birth.

Respiratory distress syndrome, as a consequence of immature lung development, is a significant risk factor for preterm birth and the major cause of early neonatal mortality and morbidity (ANZNN 2009 & Kramer 2000). Infants born very preterm (less than 32 weeks' gestation) often require respiratory support, with significant numbers requiring assisted ventilation and 9.4% remain dependent on oxygen therapy 28 days after birth and are diagnosed with chronic lung disease (ANZNN 2009). Of infants born very preterm admitted for neonatal intensive care a substantial proportion had an intraventricular haemorrhage (IVH) (21.6%) with 5.8% being Grade 3 or 4 IVH, 1.7% had periventricular leukomalacia and 4.1% of babies had retinopathy of prematurity (ANZNN 2009). Babies born preterm who survive have an increased risk of hospitalisations and a recognised risk of subsequent long-term neurodevelopmental disability including cerebral palsy (Saigal 2008 & Msall 2006). The personal and emotional costs for affected individuals and their families are high, as are the immediate and long-term monetary costs of these morbidities for parents and society (Saigal 2008, Doyle 2004 & Msall 2006).

Strategies to reduce the risk of neonatal respiratory disease for preterm birth continue to receive considerable attention (Crowther 2007, Roberts 2006 & Soll 2001). A single course of prenatal corticosteroids compared with placebo has not been shown to be effective in babies who are born more than seven days after treatment (Roberts 2006). Specifically there is no evidence for a reduction in the incidence of respiratory distress syndrome or neonatal mortality (Roberts 2006 & McLaughlin 2003) and birthweight is significantly reduced (Roberts 2006). This evidence led to the suggestion (Liggins 1972) and uptake into clinical practice within Australia (Quinlivan 1998) and other countries (Brocklehurst 1999) with minimal formal assessment, of repeating the dose of prenatal corticosteroids in the 34 to 40 per cent (Roberts 2006, McLaughlin 2002) of women who remain at risk of preterm birth seven or more days after the initial course.

Observational studies, with their inherent risk of bias, have given conflicting results, some suggesting adverse effects of repeat corticosteroids on measures of fetal growth (French 1999) and delayed development (Esplin 2000) whilst others have suggested a possible reduction in cerebral palsy.[24] Given the need for better quality evidence about the benefits and harms of repeat prenatal corticosteroids randomised clinical trials now have been reported (Aghajafari 2002, Crowther 2006, Garite 2009, Guinn 2001, Mazumder 2008, McEvoy 2002, McEvoy 2010, Murphy 2008, Peltoniemi 2007 & Wapner 2006).

CONFIDENTIAL DRAFT

Summary of systematic review of the aggregate data in 2011

It remains unclear whether repeat dose(s) or prenatal corticosteroids are beneficial. The most recent Cochrane systematic review that assesses the use of repeat prenatal corticosteroids for women at risk of preterm birth for preventing neonatal respiratory disease now includes ten trials (over 4,730 women; and 5,650 babies) with low to moderate bias (Crowther 2011).

Five of the trials were conducted in the United States of America (*Guinn 2001, McEvoy 2002, McEvoy 2010, Wapner 2006, Garite 2009*) one in Canada *Aghajafari 2002,* India (*Mazumder 2008)* and Finland (*Peltoniemi 2007)*, one in Australia and New Zealand (*Crowther 2006),* and one involved 20 countries (*Murphy 2008).*

Most trials (Aghajafari 2002, Crowther 2006, Guinn 2001, Mazumder 2008, McEvoy 2002, Wapner 2006) (6) gave repeat corticosteroids at 7 day intervals if risk of preterm birth remained, one trial (Murphy 2008) at 14 day intervals and three trials (Garite 2009, McEvoy 2010, Peltoniemi 2007) specifically targeted women for "rescue therapy" (repeat doses only given when preterm birth was considered imminent).

There was diversity in the inclusion and exclusion criteria for the ten included trials with wide variation in reasons women were at risk of preterm birth (preterm labour, preterm prelabour rupture of the membranes, antepartum haemorrhage, pre-eclampsia, growth restriction, cervical incompetence and multiple pregnancy); the gestational age women were eligible (from 24 to 34 weeks); and the time of treatment prior to expected preterm birth. All women received a single course of prenatal corticosteroids one week or more before trial entry. The type, amount and timing regime for administration of the corticosteroid given for the pre-trial course of antenatal corticosteroids varied between trials.

Treatment of women who remain at risk of preterm birth seven or more days after an initial course of prenatal corticosteroids with repeat dose(s), compared with no repeat corticosteroid treatment, reduced the risk of their infants affected by the primary outcomes *respiratory distress syndrome* (risk ratio (RR) 0.83, 95% confidence interval (CI) 0.75 to 0.91, eight trials, 3,206 infants, numbers needed to treat (NNT) 17, 95% CI 11 to 32) and *serious infant outcome* (RR 0.84, 95% CI 0.75 to 0.94, seven trials, 5094 infants, NNT 30, 95% CI 19 to 79).

Treatment with repeat dose(s) of corticosteroid was associated with a reduction in *mean birthweight* (mean difference (MD) -75.79g, 95% CI -117.63 to -33.96, nine trials, 5,626 infants). However, outcomes that adjusted birth weight for gestational age (birth weight Z scores, birth weight multiples of the median and small for gestational age) did not differ between treatment groups.

At early childhood follow-up no statistically significant differences were seen for infants exposed to repeat prenatal corticosteroids compared with unexposed infants for the primary outcomes (total deaths; survival free of any disability or major disability; disability; or serious outcome) or in the secondary outcome growth assessments.

For maximising benefit and minimising harm many questions remain. How can the potentially important benefits observed be applied to individual women who have different reasons, for and levels of risk, of preterm birth? If repeat prenatal corticosteroids are to be recommended what are the optimal gestational ages for administration, the number of repeat treatments that should be

CONFIDENTIAL DRAFT

given, and at what dose and timing? Individual patient meta-analysis of the data from the available trials may help answer these questions.

Overcoming limitations: Conducting an individual patient data meta-analysis

Analysis of thoroughly checked and updated data from individual people in all the available randomised trials has been described as the gold standard in systematic reviews.[4] Estimates of treatment effects are often different from those obtained from aggregate published data due to inclusion of additional or updated data. The methods and advantages of IPD review have been well described (Stewart 2002, Cochrane Collaboration IPD Group).

An integral component of conducting this IPD meta-analysis is the formation of an international collaborative group of trialists where all researchers endorse the IPD protocol and provide data from their trials. This generates additional benefits that include:

- more complete identification of trials and of trial details,
- compliance with standard definitions, provision of missing data on characteristics of trials, all women who were randomised and their babies, and outcomes,
- more balanced interpretation, endorsement and global dissemination of results, and
- better clarification and consensus on future research needed with the opportunity for ongoing international collaborations (Stewart 1995).

Objectives

To assess, using individual patient data meta-analyses, whether the effects of repeat prenatal corticosteroid treatment given to women at risk of preterm birth on important clinical outcomes both short-term and long-term and whether treatment effects differ in a clinically meaningful way between important prespecified patient-level characteristics.

Research Questions

For maximising benefit and minimising harm the main research questions to be addressed in this review are:

a) *Are repeat prenatal corticosteroids more effective in some women by reason of their risk of preterm birth?*
b) *If the use of repeat prenatal corticosteroids is recommended what is the best gestational age to maximise benefit?*
c) *What dose, number of repeat doses and timing prior to birth is optimal?*
d) *What is the minimal effective dose of repeat prenatal corticosteroids?*
e) *Is a single rescue steroid dose effective?*

Methods

Inclusion and exclusion criteria for the studies

The inclusion and exclusion criteria for the types of study design, participants and interventions are listed below. Eligibility of trials will be assessed independently and unblinded for author and journal by two members of the PRECISE IPD Project Team. Any differences in opinion regarding eligibility will

CONFIDENTIAL DRAFT

be resolved by discussion. If individual patient data are unavailable from any eligible trial, the trial will still be included in the review and aggregate data will be used for sensitivity analyses wherever possible.

Study design: Studies, published or unpublished, will be included if they were randomised trials with adequate allocation concealment and report data on one or more of the pre-stated outcomes. Quasi-random study designs will not be included.

Types of participants: Women considered at risk of preterm birth who have already received a single course of prenatal corticosteroid seven or more days previously.

Types of interventions: Corticosteroid administered to the women intravenously, intramuscularly or orally, compared with either placebo or no placebo. Trials in which the fetus receives corticosteroids directly will not be included.

Search Strategy to identify potential trials

The trials included in the updated Cochrane review of aggregate data about repeat prenatal corticosteroids will be eligible for inclusion in the IPD analysis as well as any more trials found through a repeat of the search. The search will use the same search strategy and databases (Medline, Embase) to find relevant randomised controlled trials. This will include a search of the *The Cochrane Central Register of Controlled Trials* using the terms [repeat or multiple] and [antenatal or prenatal] and [corticosteroid* or steroid* or glucocorticoid* or betamethason* or dexamethason* or hydrocortison*]. Furthermore, experts in the field and trialists will be asked to report on any unfound or unpublished trials.

Data collection and management

Data will be collected on all women randomised and on women who were excluded after randomisation and will include basic identification of the women, coded for anonymity, (date of birth, centre identification); baseline data for descriptive purposes and analyses (reason at risk of preterm birth, gestational age at trial entry, plurality of the pregnancy, expected date of delivery); details of the intervention given (date of randomisation, allocated intervention, type and dose of corticosteroid given, interval between treatments, whether re-treatment given and amount); outcomes of interest listed below sufficient to allow planned analyses.

Trialists can provide the individual patient data in any format by encrypted, electronic transfer where possible or other means as needed. The individual trial data will be recoded according to the agreed protocol. Data transformation to the new format and coding system will be done by the trialists or by the PRECISE investigators' team. Only authorized personnel (members of the Data Management Team) will have access to the data. However, Collaborators will continue to have control over how data from their trial is used. Recoded data will be stored in a secure custom designed database. The data will not be used for any other purpose without permission of the collaborators.

The data will be checked with respect to range, internal consistency, missing or extreme values, errors and consistency with published reports. Trial details such as randomisation methods and intervention details will be crosschecked against published reports, trial protocols and data

CONFIDENTIAL DRAFT

collection sheets. Inconsistencies or missing data will be discussed with the individual trialists and attempts will be made to resolve any problems by consensus. Each trial will be analysed individually, and the resulting analyses and trial data will be sent to the trialists for verification.

Data items to be collected

Trial level information:

1. Dates the trial opened and closed accrual
2. Number of patient randomised
3. Informed consent procedures
4. Methods of random allocation
5. Stratification factors
6. Methods of allocation concealment
7. Blinding procedures for outcome assessment
8. Purpose repeat corticosteroid treatment given (prophylaxis against preterm birth, 'rescue therapy' when preterm birth is imminent, other)
9. Details of the planned intervention in the experimental arms
    a. Type of repeat prenatal corticosteroid treatment given
    b. Number of repeat doses planned to be given
    c. Minimum planned interval between the initial corticosteroid and the repeat dose
    d. Minimum planned interval between repeat steroid treatments
    e. Planned dose of corticosteroid to be given per repeat treatment
    f. Planned dose of repeat steroid drug exposure per week
    g. Total drug exposure planned
10. Details of the planned intervention in the control arm

Participant-level information: maternal characteristics at study entry

1. Unique identification coded for anonymity
2. Maternal age
3. Parity
4. Ethnicity
5. Public or private patient
6. Previous obstetric history
7. Reason the woman was considered to be at risk of preterm birth (such as preterm labour, the presence or absence of ruptured membranes, antepartum haemorrhage, pre-eclampsia, growth restriction, suspected fetal jeopardy, cervical incompetence, maternal disease and multiple pregnancy)
8. Pretrial treatment with corticosteroids (gestation dose given, corticosteroids used, doses regimen)
9. Reason repeat prenatal corticosteroid treatment was considered (prophylaxis against preterm birth or 'rescue therapy' when preterm birth is imminent or other)
10. Number of babies in-utero
11. Gestational age when repeat prenatal corticosteroid treatment was started

CONFIDENTIAL DRAFT

12. Time prior to birth repeat prenatal corticosteroid treatment was given

Participant-level information: data on actual study intervention relating to regimens

Actual treatment received (drug frequency, timing and doses):

- Type of repeat prenatal corticosteroid treatment given
- Number of repeat doses actually given
- Minimum actual interval between the initial corticosteroid and the study repeat dose
- Minimum actual interval between repeat steroid treatments
- Actual dose of corticosteroid given per repeat treatment
- Actual dose of repeat steroid drug exposure per week
- Total actual drug exposure
- Rescue treatment vs weekly treatment

Participant-level information: data on neonatal outcomes

1. Unique baby identification and mother identification coded for anonymity
2. Date and time of birth
3. Gestational age at birth
4. Gender
5. Birth weight, length, head circumference
6. Apgar score at 1' and 5' minutes
7. Neonatal complications/status
8. Mortality and age at death
9. Cause of death
10. Childhood follow-up assessments

Planned analyses

A detailed statistical analysis plan will be discussed and agreed upon by all the PRECISE Collaborators prior to any data analyses.

Analyses will aim to be of all women ever randomised and will be based on intention to treat whereby outcomes with data available will be included according to the groups to which the women were randomised.

A one-staged approach to analysis will be taken so that the individual patient data from all eligible trials are included in a single model (Whitehead 2002). Fitting a single model for each outcome will enable the different trials and multiple births to be adjusted for within the model. Binary outcomes will be analysed using appropriate generalised estimating equations for binary data and continuous outcomes will be analysed using linear models. Accounting for correlation between outcomes from multiple births will be incorporated as random effects.

CONFIDENTIAL DRAFT

Inferences from subgroup analyses will be conducted by examining the significance of the test for the treatment by subgroup interaction term (Rothwell 2005). For each subgroup analysis a model will be fitted which includes the treatment by subgroup of interest interaction term and if this term is not statistically significant then the only appropriate estimate of treatment effect is the overall estimate, not the estimates within individual groups.

1. Outcomes to be analysed

Outcomes have been chosen to be most representative of the clinically important measures of effectiveness and safety, including serious outcomes, for the women and their babies.

Primary Outcomes

For the infants:

- **serious adverse outcome** defined by the PRECISE Collaboration as death (fetal, neonatal or infant); severe respiratory disease; severe IVH (grade 3 & 4); cystic periventricular leukomalacia, chronic lung disease; necrotising enterocolitis; severe retinopathy of prematurity);
- **birthweight.**

For the children:

- **death (fetal, neonatal or later death up to the time of follow up);**
- **death or any neurological disability** (however defined by trialists and may include developmental delay or intellectual impairment [developmental quotient or intelligence quotient less than one SD below the mean], cerebral palsy [abnormality of tone with motor dysfunction], blindness [corrected visual acuity worse than 6/60 in the better eye], or deafness [hearing loss requiring amplification or worse], at follow up later in childhood).

For the women:

- **maternal sepsis** (defined as chorioamnionitis (however defined by trialists); or pyrexia after trial entry requiring the use of antibiotics; or puerperal sepsis (however defined by trialists); or intrapartum fever requiring the use of antibiotics; postnatal pyrexia (however defined by trialists)].

Secondary Outcomes

Secondary outcomes for the infants and children will be key outcomes available from the trials to be included in the IPD.

For the infants:

- serious adverse outcome (defined by the trialists as death fetal, neonatal or later death and may include respiratory distress syndrome; severe lung disease; chronic lung disease; intraventricular haemorrhage; patent ductus arteriosus requiring treatment, neonatal encephalopathy; retinopathy of prematurity);

CONFIDENTIAL DRAFT

- gestational age at birth (preterm birth less than 37 weeks, very preterm birth less than 34 weeks, extremely preterm birth less than 28 weeks);
- interval between trial entry and birth;
- small-for-gestational age;
- head circumference at birth;
- length at birth;
- placental weight;
- Apgar score less than seven at five minutes;
- use of respiratory support (defined as mechanical ventilation or continuous positive airways pressure (CPAP) or other respiratory support);
- duration of respiratory support;
- use of oxygen supplementation;
- duration of oxygen supplementation;
- use of surfactant;
- air leak syndrome;
- use of inotropic support;
- use of nitric oxide for respiratory support;
- any intraventricular haemorrhage (IVH)
- severe IVH
- cystic periventricular leukomalacia;
- early neonatal infection (however defined by trialists);
- proven neonatal infection while in the neonatal intensive care unit;
- admission to neonatal intensive care unit;
- necrotising enterocolitis (however defined by the trialists);
- patent ductus arteriosus (however defined by the trialists);
- retinopathy of prematurity;
- use of postnatal corticosteroids;
- neonatal blood pressure (systolic, diastolic and mean arterial blood pressure);
- growth assessments at primary hospital discharge (weight, head circumference, length);

CONFIDENTIAL DRAFT

- hypothalamo/pituitary/adrenal (HPA) axis suppression (however defined by the trialists).

For the children (follow up):

- **neurologic impairments**
    - cerebral palsy (CP) (categorised as nil, mild, moderate or severe, as defined by the trialists);
    - developmental delay or intellectual impairment (categorised as nil, mild, moderate or severe, by trialists);
    - blindness;
    - deafness;
    - gross motor dysfunction (defined as mild, moderate or severe, by trialists or by the Gross Motor Classification System [score 0-5], if available);
    - psychomotor dysfunction (categorised as nil, mild (<85), moderate (<70) or severe (<55) by the Psychomotor Development Index (PDI)).
- **any neurological disability** (defined as developmental delay or intellectual impairment [developmental quotient or intelligence quotient less than one SD below the mean], cerebral palsy [abnormality of tone with motor dysfunction], blindness, or deafness, at follow up later in childhood);
- **major neurologic disability** (defined as any moderate or severe neurological impairment)
- **survival free of major neurological disability** (alive and without major disability, however defined by trialists);
- **growth assessments at childhood follow up** (Z-scores for weight, head circumference, length);
- **child behaviour** (however defined by trialists);
- **child temperament**;
- **respiratory disease** (however defined by trialists);
- **blood pressure.**

For the women:

- **death;**
- **chorioamnionitis** (however defined by trialists)**;**
- **puerperal sepsis** (however defined by trialists)**;**
- **pyrexia after trial entry requiring the use of antibiotics;**
- **intrapartum fever requiring the use of antibiotics;**
- **postnatal pyrexia** (however defined by trialists)**.**
- **admission to intensive care unit;**
- **prelabour rupture of the membranes after trial entry;**
- **hypertension** (variously defined by the trialists);
- **mode of birth;**

CONFIDENTIAL DRAFT

- **postpartum haemorrhage;**
- **breastfeeding after hospital discharge;**
- **postnatal depression;**
- **side-effects of corticosteroid therapy** (including gastrointestinal upset, glucose intolerance, insomnia, pain at the injection site, bruising at the injection site, Cushing appearance)**;**
- **discontinuation of corticosteroid therapy because of maternal side-effects.**

Use of health services:

- length of antenatal hospitalisation for the women;
- length of postnatal hospitalisation for the women;
- maternal admission to intensive care unit;
- admission to and length of stay in neonatal intensive care unit;
- length of neonatal hospitalisation.

2. Planned subgroup analyses

Where sufficient data exist, subgroup comparisons will be conducted using the five infant, child and maternal primary outcomes. Any differences in treatment effect between subgroups will be assessed by testing a treatment by subgroup interaction term within the model.

Subgroups

a. Trial-level characteristics

1. Type of repeat prenatal corticosteroid treatment given (betamethasone or dexamethasone).
2. Number of repeat doses planned to be given.
3. Minimum planned interval between the initial corticosteroid and the first repeat dose.
4. Minimum planned interval between repeat steroid treatments.
5. Planned dose of corticosteroid per repeat treatment.
6. Planned dose of repeat steroid drug exposure per week.

b. Participant-level characteristics

1. Reason the woman was considered to be at risk of preterm birth (preterm labour, the presence or absence of ruptured membranes, antepartum haemorrhage, pre-eclampsia, growth restriction, cervical incompetence and multiple pregnancy)
2. Purpose repeat corticosteroid treatment given (prophylaxis against preterm birth, ‘rescue therapy’ when preterm birth is imminent, other)
3. Number of babies in-utero
4. Gestational age when first repeat prenatal corticosteroid treatment was given (<26; 26 to 27; 28 to 29; 30 to 31; 32 to 33 completed weeks at randomisation)

CONFIDENTIAL DRAFT

5. Time prior to birth last dose of repeat prenatal corticosteroid treatment was given (<1 day; <2 days; 2 to 4; 4 to <7; 7 to <10; 10 to <14; ≥14 days)
6. Number of repeat doses actually given.
7. Minimum actual interval between the initial corticosteroid and the first repeat dose.
8. Minimum actual interval between repeat steroid treatments.
9. Actual dose of corticosteroid given per repeat treatment.
7. Actual dose of repeat steroid drug exposure per week.
8. Total actual drug exposure (12 mg; 24 mg; 36 mg; 48 mg; 60 mg; 72 mg; 84 mg; 96 mg; 108 mg; 120 mg; 132 mg; 144 mg)

3. Planned sensitivity analyses

a) To assess whether the results are robust to trial design and quality, the following sensitivity analyses will be performed:

- Exclusion of trials with small sample size (< 100 study patients)
- Trials with high rate of exclusions (20% or more)
- Inclusion of aggregate data from trials where individual patient data are unavailable.

Ethical considerations

Participants in the individual trials have previously given informed consent to participate in their respective trial. The data for this project are to be used for the purpose for which they were originally collected and are available through an agreement between all trialists of the collaborative group. These trialists remain the custodian of their original individual trial data at all times.

Project management

For the purpose of this project, an international Collaborative Group, the PRECISE Collaboration, has been formed. The PRECISE Collaboration consists of groups with specific responsibilities and tasks:

1. The PRECISE IPD Project Team
The PRECISE IPD Project Team is the Steering Group which is responsible for the project's management decisions and the daily management of the Collaboration. The Project Team's tasks are to design the project's protocol and analysis plan, organize the PRECISE International IPD Study Group Meetings and act as a liaison between all the members of the PRECISE International IPD Study Group. Membership: C Crowther[1] (chair), P Middleton[1] (epidemiologist), L Askie[2] (responsible for Data Management Team), L Doyle[3] (advise on paediatric aspects), T Bubner[1] (project coordinator) and the PRECISE coordinating statistician. The PRECISE IPD Project Team will meet regularly every 2 to 4 months, usually by teleconference.

[1]Australian Research Centre for Health of Women and Babies, Discipline of Obstetrics and Gynaecology, The University of Adelaide.

[2]NHMRC Clinical Trials Centre, University of Sydney, Sydney, Australia.

CONFIDENTIAL DRAFT

[3]Department of Obstetrics and Gynaecology, University of Melbourne.

2. The PRECISE International IPD Study Group
Members of the PRECISE International IPD Study Group will be representatives of the eligible trials. For each trial, the first author will be invited to become a member of the Collaboration. If considered necessary or if there was no response from the first author, other investigators from the trials may be contacted (data manager, statistician). In order to keep the PRECISE International IPD Study Group updated, authors of new trials or previously unidentified trials will be contacted and invited to join the Collaboration in the course of the project.

3. Data Management Centre
The data management centre will be located at ARCH in Adelaide, Australia, and will be coordinated by C Crowther, Chair of the PRECISE IPD Project Team. The PRECISE coordinating statistician will lead the AMICABLE Statistical Team and coordinate the analyses. The data management centre will be responsible for the storage and analyses of the project data.

The PRECISE International IPD Study Group Meetings
Collaborative group face to face meetings will be organised at least twice during the study. Members of all the trials will be invited to attend those meetings. The meetings will be scheduled, if possible, in conjunction with international conferences. During those meetings, various aspects of the project will be discussed with all the collaborators, such as the project's design and conduct, the analysis plan, and the interpretation and reporting of the results. The final PRECISE Collaborators' meeting is scheduled for 2012.

Funding

The National Health and Medical Research Council (NHMRC) through a project grant is funding the PRECISE IPD Study. This funding will support the collection of the individual patient data by the original investigators and to organise the Collaborators' meetings. The NHMRC is not involved in any other aspect of the project, such as the design of the project's protocol and analysis plan, the collection and the analyses of the project's data, or the interpretation and the publication of the study results.

Publication Policy

The final results of the study will be presented to the collaborators for discussion. The main manuscript will be prepared by the PRECISE IPD Project Team and circulated to the other members of the Collaboration for comment and revision. The revised draft paper will be circulated for final comment and agreement prior to publication. PRECISE publications arising from these data will be authored with specific named authors and on behalf of the PRECISE Collaboration as a whole. The names of all other participating Collaborators will be acknowledged in the appropriate section of the manuscript.

Conclusion

The recently updated meta-analysis showed that the short term benefits seen for babies support the use of repeat dose(s) of prenatal corticosteroids for women who have received an initial course of prenatal corticosteroids seven of more days previously and who remain at risk of preterm birth.

CONFIDENTIAL DRAFT

For maximising benefit and minimising harm many questions remain. Are repeat prenatal corticosteroids more effective in some women by reason of their risk of preterm birth? If the use of repeat prenatal corticosteroids is recommended what is the best gestational age to use for benefit? What dose, number of repeat doses and timing prior to birth is optimal?

The best way to answer these remaining questions is to utilize existing individual patient data from all women and babies enrolled in these trials. This approach has been described as the 'gold standard' of systematic review methodology as it allows for more powerful and flexible analysis of both subgroups and outcomes. The PRECISE Collaboration has been formed to undertake a meta-analysis based on individual patient data, to answer these important clinical questions. Provision of data by the participating Collaborators commenced in 2010 and results will be ready for presentation in 2013.

CONFIDENTIAL DRAFT

References

1. **ANZNN** (Australian & New Zealand Neonatal Network) Report of the Australian and New Zealand Neonatal Network 2006. Sydney: ANZNN, 2009.
2. **Crowther CA**, McKinlay C, Middleton PM, Harding JE. Repeat doses of prenatal corticosteroids for women at risk of preterm birth for improving neonatal health outcomes. Cochrane Database of Systematic Reviews 2011:3. In press.
3. **Stiles AD.** Prenatal corticosteroids – Early gain, long term questions. New England Journal of Medicine 2007;357:1248-1250.
4. **Chalmers I.** The Cochrane Collaboration: preparing, maintaining, and disseminating systematic reviews of the effects of health care. Ann. N. Y. Acad. Sci. 1993;703:156-63; discussion 163-165.
5. **Laws P** & Sullivan EA. Australia's mothers and babies 2007. Perinatal statistics series no. 23. Cat. no. PER 48. Sydney: AIHW National Perinatal Statistics Unit, 2009.
6. **Saigal S** & Doyle L. An overview of mortality and sequelae of preterm birth from infancy to adulthood. Lancet 2008;371:261-269.
7. **Doyle LW** and the Victorian Infant Collaborative Study Group. Evaluation of neonatal intensive care for extremely LBW infants in Victoria over two decades. Pediatrics 2004;113:510-514.
8. **Doyle LW** for the Victorian Infant Collaborative Study Group. Changing availability of neonatal intensive care for extremely LBW infants over two decades. Med J Aust 2004;181:136-139.
9. **Msall M.** The panorama of cerebral palsy after very and extremely preterm birth: evidence and challenges. Clin Perinatol 2006;33:269-284.
10. **Kramer M,** Demissie K, Yang H, Platt RW, Sauvé R, Liston R. Contribution of preterm birth to mortality. JAMA 2000;843:849.
11. **NHMRC.** The NHMRC Strategic Plan 2007-2009: Working to build a healthy Australia. Canberra: Australian Government, 2007.
12. **Roberts D,** Dalziel S. Antenatal corticosteroids for accelerating fetal lung maturation for women at risk of preterm birth. Cochrane Database Syst Rev 2006:3. Art. No.: CD004454. DOI: 10.1002/14651858.CD004454.pub2.
13. **Soll R,** Morley C. Prophylactic versus selective use of surfactant in preventing morbidity and mortality in preterm infants. Cochrane Database Syst Rev. 2001: 2. Art. No.: CD000510. DOI: 10.1002/14651858.CD000510.
14. **Dalziel S,** Walker N, Parag V, Mantell C, Rea H, Rodgers A, Harding JE. Cardiovascular risk factors after antenatal exposure to betamethasone: 30 year follow-up. Lancet 2005;365:1856-1862.
15. **NIH Consensus Development Panel**. Antenatal corticosteroids revisted: repeat courses. National Institutes of Health Consensus development conference statement. Obstet Gynecol. 2000;98:144-150.
16. **Quinlivan JA**, Evans SF, Dunlop SA Beazley LD, Newnham JP. Use of corticosteroids by Australian obstetricians - a survey of clinical practice. Aust NZ J Obstet Gynaecol 1998;38(1):1-7.
17. **Foix-L'Helias L**, Marret S, Ancel , Marchand L, Arnaud C, Fresson J, Picaud J, Rozé J, Theret B, Burguet A, Larroque B, Kaminski M; the EPIPAGE Study Group. Impact of antenatal corticosteroids on mortality cerebral lesions and 5-year neurodevelopmental outcomes. BJOG 2008;115(2):275-282.
18. **McLaughlin K**, Crowther C, Walker N, Harding J. Effects of a single course of corticosteroids given more than 7 days before birth: a systematic review. Aust N Z J Obstet Gynaecol. 2003;43:101-106.
19. **Liggins G,** Howie R. A controlled trial of antepartum glucocorticoid treatment for prevention of the respiratory distress syndrome in premature infants. Pediatrics 1972;50:515-525.

CONFIDENTIAL DRAFT

20. **Brocklehurst P,** Gates S, McKenzie-McHarg, Alfirevic A, Chamberlain G. Are we prescribing multiple courses of antenatal corticosteroids? A survey of practice in the UK. BJOG 1999;106(9):977-979.
21. **McLaughlin K**, Crowther CA, Vigneswaran P, Hancock E, Willson K. Who remains undelivered more than seven days after a single course of prenatal corticosteroids and gives birth at less than 34 weeks? Aust N Z J Obstet Gynaecol. 2002 Oct;42(4):353-357.
22. **French N**, Hagan R, Evans S, Godfrey M, Newnham J. Repeated antenatal corticosteroids: size at birth and subsequent development. Am J Obstet Gynecol. 1999;180:114-121.
23. **Esplin M**, Fausett M, Smith S, Oshiro B, Porter T, Branch D, Varner M. Multiple courses of antenatal steroids associated with a delay in long-term psychomotor development in children with birth weight <1500 grams. Am J Obstetrics Gynecol. 2000;182(1 Pt 2):S24
24. **French N**, Hagan R, Evans S, Mullan A, Newnham J. Repeated antenatal corticosteroids: effects on cerebral palsy and childhood behaviour. American J Obstet Gynecol. 2004;190:588-595.
25. **Aghajafari F**, Murphy K, Ohlsson A, Amankwah K, Matthews S, Hannah M. Multiple versus single courses of antenatal corticosteroids for preterm birth: a pilot study. J Obstet Gynaecol Can: JOGC. 2002;24(4):321-329.
26. **Crowther C,** Haslam R, Hiller J, Doyle L, Robinson J, for the ACTORDS Study Group. Neonatal respiratory distress syndrome after repeat exposure to antenatal corticosteroids: a randomised controlled trial. Lancet. 2006:367:1913-1919.
27. **Garite T,** Kurtzman J, Maurel K, Clark R for the Obstetrix Collaborative Research Network. Impact of a 'rescue course' of antenatal corticosteroids: a multicenter randomized placebo-controlled trial. Am J Obstet Gynecol 2009;200:248.e1-248.e9.
28. **Guinn D,** Atkinson M, Sullivan L, Lee M, MacGregor S, Parilla B, Davies J, Hanlon-Lundberg K, Simpson L, Stone J, Wing D, Ogasawara K, Muraskas J. Single vs weekly courses of antenatal corticosteroids for women at risk of preterm delivery. JAMA. 2001;286(13)1581-1587.
29. **Mazumder P,** Dutta S, Kaur J, Narang A. Single versus multiple courses of antenatal betamethasone and neonatal outcome: a randomized controlled trial. Indian Pediatr 2008;45:650-652.
30. **McEvoy C,** Bowling S, Williamson R, Lozano D, Tolaymat L, Izquierdo L, Maher J, Helfgott A. The effect of a single remote course versus weekly courses of antenatal corticosteroids on functional residual capacity in preterm infants: a randomized trial. Pediatrics 2002;110:280-284.
31. **Murphy K,** Hannah M, Willan A, Hewson S, Ohlsson A, Kelly E, for the MACS Collaborative Group. Multiple courses of antenatal corticosteroids for preterm birth (MACS): a randomised controlled trial. Lancet 2008;372:2143-2151.
32. **Peltoniemi O,** Kari M, Tammela O, Lehtonen L, Marttila R, Halmesmaki E, Jouppila P, Hallman M; Repeat Antenatal Betamethasone Study Group. Randomized trial of a single repeat dose of prenatal betamethasone treatment in imminent preterm birth. Pediatrics 2007;119:2:290-298.
33. **Wapner R,** Sorokin Y, Thom E, Johnson F, Dudley D, Spong ,C, Peaceman AM, Leveno KJ, Harper M, Caritis SN, Miodovnik M, Mercer B, Thorp JM, Moawad A, O'Sullivan MJ, Ramin S, Carpenter MW, Rouse DJ, Sibai B, Gabbe SG; National Institute of Child Health and Human Development Maternal Fetal Medicine Units Network. Single versus weekly courses of antenatal corticosteroids:evaluation of safety and efficacy. Am J Obstet Gynecol. 2006;195:633-642.
34. **Crowther CA**, Doyle LW, Haslam RR, Hiller JE, Harding JE, Robinson JS for the ACTORDS Study Group. Outcome at 2 years of age after repeat doses of antenatal corticosteroids. New England Journal Medicine 2007;357:1179-1189.
35. **Wapner R**, Sorokin Y, Mele L, Johnson F, Dudley D, Spong Peaceman AM, Leveno KJ, Malone F, Caritis SN, Mercer B, Harper M, Rouse DJ, Thorp JM, Ramin S, Carpenter MW, Gabbe SG; National Institute of Child Health and Human Development Maternal-Fetal Medicine Units Network. Long-term outcomes after repeat doses of antenatal cortiocosteroids. New England Journal Medicine 2007;35:1190-1198.

CONFIDENTIAL DRAFT

36. **Peltoniemi O**, Kari, M, Lano A, Yliherva A, Puosi R, Lehtonen L, Hallman M. Two-year follow-up of a randomized trial with repeated antenatal betamethasone. Arch Dis Child. Fetal Neonatal Ed. 2009;94(6):F402-6.
37. **Bonanno C,** Wapner R. To rescue or not: that is the question. Am J Obstet Gynecol 2009:200(3);217-218.
38. **Newnham JP,** Jobe AH. Should we be prescribing repeated courses of antenatal corticosteroids? Seminars in Fetal and Neonatal Medicine 2009;14:157-163.
39. **Stewart L,** Tierney J. To IPD or not to IPD? Advantages and disadvantages of systematic reviews using individual patient data. Eval Health Prof 2002;25(1):76-97.
40. **Cochrane Collaboration IPD Group**, Stewart L, Tierney J, Clarke M. Chapter 19 Reviews of individual patient data. In: Higgins JPT, Green S (editors). Cochrane Handbook for Systematic reviews of Interventions Version 5.0.0 [updated February 2008]. The Cochrane Collaboration, USA: John Wiley and Sons Ltd, 2008.
41. **Brocklehurst P**, Gates S, Johnson A. Effects of multiple courses of antenatal steroids are uncertain [letter]. BMJ 2000;321:347.
42. **Palisano R**, Rosenbaum P, Walter S, Russell D, Wood E, Galuppi B. Development and reliability of a system to classify gross motor function in children with cerebral palsy. Dev Med Child Neurol 1997;39:214-223.
43. **Stewart L,** Clarke M. Practical methodology of meta-analyses (overviews) using updated individual patient data. Cochrane Working Group. Statistical Medicine 1995;14(19):2057-2079.
44. **Lambert P**. A comparison of summary patient-level covariates in meta-regression with individual patient data meta-analysis. J Clin. Epidemiol. 2002;55(1):86-94.
45. **Whitehead A**. Meta-analysis of controlled clinical trials. USA: John Wiley & Sons, 2002.
46. **Rothwell PM**. Subgroup analysis in randomised controlled trials: Importance, indications, and interpretation. Lancet 2005; 365: 176-86.
47. **Wang R**, Lagokos S, Ware J, Hunter D, Drazen J. Statistics in Medicine- Reporting of subgroup analyses in clinical trials New England Journal Medicine 2007; 357:21.

CONFIDENTIAL DRAFT
